# Anti‐pyroptotic function of TGF‐β is suppressed by a synthetic dsRNA analogue in triple negative breast cancer cells

**DOI:** 10.1002/1878-0261.12890

**Published:** 2021-01-04

**Authors:** Yusuke Tamura, Masato Morikawa, Ryo Tanabe, Kohei Miyazono, Daizo Koinuma

**Affiliations:** ^1^ Department of Molecular Pathology Graduate School of Medicine The University of Tokyo Japan

**Keywords:** polyI:C, pyroptosis, RLR, TGF‐β, TNBC

## Abstract

Development of innovative therapeutic modalities would address an unmet clinical need in the treatment of triple negative breast cancer (TNBC). Activation of retinoic acid‐inducible gene‐I (RIG‐I)‐like receptors (RLRs) such as melanoma differentiation‐associated gene 5 (MDA5) and RIG‐I in cancer cells is suggested to suppress tumor progression by inducing cell death. Transfection of polyI:C, a conventionally used synthetic double‐stranded RNA (dsRNA) analogue that activates RLRs, has been evaluated in clinical trials. However, detailed mechanisms of tumor suppression by RLRs, especially interactions with other signaling pathways, remain elusive. Here, we showed that transfection of polyI:C suppressed transforming growth factor‐β (TGF‐β) signaling in a MDA5‐ and RIG‐I‐dependent manner. We found that suppression of TGF‐β signaling by polyI:C promoted cancer cell death, which was attenuated by forced expression of constitutively active Smad3. More detailed analysis suggested that cell death by polyI:C transfection exhibited characteristics of pyroptosis, which is distinct from apoptosis. Therapeutic efficacy of polyI:C transfection was also demonstrated using a mouse model. These results indicated that intratumor administration of polyI:C and related dsRNA analogues may be promising treatments for TNBC through inhibition of the anti‐pyroptotic function of TGF‐β.

## Graphical abstract

The present study provides a new perspective of the treatment of triple negative breast cancer (TNBC) through the intracellular administration of polyI:C, a ligand for retinoic acid‐inducible gene‐I‐like receptors. PolyI:C induced the pyroptosis of TNBC cells, which was enhanced by its inhibitory role on the transforming growth factor‐β (TGF‐β)‐regulated anti‐pyroptotic functions.

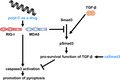

**Abbreviations**
CCLE: Cancer Cell Line Encyclopedia
cGAS: cyclic GMP‐AMP synthase
dsRNA: double‐stranded RNA
EMT: epithelial–mesenchymal transition
gRNAs: guide RNAs
GSDMD: gasdermin D
GSDME: gasdermin E
GSEA: gene set enrichment analysis
IFN: interferon
IRF3: interferon regulatory factor 3
LDH: lactate dehydrogenase
LPS: lipopolysaccharide
MDA5: melanoma differentiation‐associated gene 5
PARP: poly (ADP‐ribose) polymerase
PD‐L1: programmed death‐ligand 1
PI: propidium iodide
PRRs: pattern recognition receptors
RIG‐I: retinoic acid‐inducible gene‐I
RLRs: RIG‐I‐like receptors
STING: stimulator of interferon genes
TBK1: TANK‐binding kinase 1
TGF‐β: transforming growth factor‐β
TNBC: triple negative breast cancer

## 1 Introduction

Triple negative breast cancer (TNBC) is one of the most aggressive types of cancer and constitutes ~ 16% of invasive breast cancer cases [1, 2]. Although conventional cytotoxic chemotherapy was the only option for treating TNBC for many years, molecular target therapies such as poly (ADP‐ribose) polymerase (PARP) inhibitors for patients with BRCA mutations and antibodies against programmed death‐ligand 1 (PD‐L1) or PD‐1 for PD‐L1‐positive patients have been approved for use in recent years [3]. Because PD‐L1 expression tends to be higher in patients with TNBC than those in other subtypes of breast cancer, immune checkpoint inhibitors are expected to have higher potential for treatment of TNBC patients [4, 5].

Activating innate immune signaling in cancer cells, such as by intratumoral injection of ligands, is currently proposed to be an interesting strategy to combat cancer cells and is expected to be applied concomitantly with immune checkpoint inhibitors [6, 7]. The group of receptors involved in activating innate immune signaling are called pattern recognition receptors (PRRs), which include Toll‐like receptors (TLRs) and nucleotide‐binding oligomerization domain (NOD)‐like receptors (NLRs). PRRs recognize pathogenic components to protect hosts from pathogens or molecules released from damaged cells to notify surrounding cells of danger. Among PRRs, activation of retinoic acid‐inducible gene‐I (RIG‐I)‐like receptors (RLRs), which include melanoma differentiation‐associated gene 5 (MDA5) and RIG‐I in cancer cells, is suggested as beneficial strategy in the treatment of TNBC due to the fact that the RIG‐I gene is rarely mutated or deleted [8]. RLRs can recognize cytosolic double‐stranded RNA (dsRNA) to trigger the formation of a complex with mitochondrial antiviral signaling protein (MAVS). The complex then induces interferon (IFN) by phosphorylation of interferon regulatory factor 3 (IRF3) and informs the surrounding cells of a virus infection [9, 10]. RLR agonists are also expected to be applicable to cancer treatment by inducing tumor cell death and increasing the immunogenicity of tumors by upregulating IFNs or proinflammatory cytokines [7, 11]. This is supported by findings that some RIG‐I and MDA5 agonists show promising effects in mouse tumor models when administered by intratumoral transfection [12]. Intratumoral transfection of these agonists is also being evaluated in clinical trials of aggressive solid tumors, including TNBC.

Transforming growth factor‐β (TGF‐β) signaling is one of the most important pathways in cancers. Upon TGF‐β binding, a heterotetrameric complex of type I and type II TGF‐β receptors (TβRI and TβRII) is formed to induce the phosphorylation of downstream components Smad2 and Smad3. Activated Smad2 and Smad3 then form a complex with Smad4, translocate to the nucleus, and trigger dramatic genome‐wide changes in transcription [13]. It is important to note that TGF‐β is believed to have both tumor‐suppressive and tumor‐promoting functions [14, 15, 16]. Antiproliferative effects and the promotion of cell death are tumor‐suppressive facets of TGF‐β, and cancers often evade TGF‐β signaling by mutating TGF‐β receptors or Smads [16]. On the other hand, with respect to the tumor‐promoting aspect of TGF‐β, cancers may acquire significant benefits from this signaling pathway. There are a number of reports that focus on the protumorigenic function of TGF‐β using mammary carcinoma cells to investigate the cooperation of mutant RAS proto‐oncogenes with TGF‐β in the induction of epithelial–mesenchymal transition (EMT) or enhanced cellular invasion, the stabilization of mammalian target of rapamycin signaling to retain the stemness of cells by prolonged TGF‐β exposure, and the collaboration with jun B proto‐oncogene (JUNB) to enhance cell invasion [17, 18, 19, 20]. Likewise, the promotion of migration, invasion, bone metastasis, and cell survival are characteristics of TGF‐β in TNBC [21, 22]. It is also reported that TGF‐β reduces the expression of major histocompatibility class I and suppresses CD8+ T‐cell‐mediated killing of other types of cancers [23]. Taken together, there appears to be more potent characteristics of protumorigenic function of TGF‐β signaling in TNBC, and suppression of TGF‐β signaling is a well‐founded strategy to combat TNBC.

Regarding the interaction between RLR signaling and TGF‐β signaling, a previous report revealed that IRF3 activated by dsRNA ligand physically interacts with Smad3 to inhibit its binding to TβRI, which leads to the attenuation of Smad3 phosphorylation [24]. However, the effect of attenuating TGF‐β signaling by RLR ligands on cancer cells is not well characterized, especially in context of the protumorigenic function of TGF‐β signaling. Furthermore, the previous report did not evaluate dsRNA as a therapeutic ligand for cancer [24].

In the present study, we chose the synthetic dsRNA analogue polyI:C as an RLR agonist, which is commonly and classically used experimentally and has been evaluated in clinical trials of aggressive solid tumors [12]. We found that polyI:C suppressed TGF‐β signaling in TNBC cells in an MDA5‐ and RIG‐I‐dependent manner, which led to attenuation of the prosurvival function of TGF‐β signaling. We also explored the characteristics of cell death induced by polyI:C in TNBC cells and revealed a novel role for TGF‐β as an anti‐pyroptotic factor. These findings provide support for the efficacy of polyI:C as a promising drug for the treatment of TNBC partly mediated by the inhibition of TGF‐β signaling.

## 2 Materials and methods

### 2.1 Cell culture

Hs578T (HTB‐126) and BT‐549 (HTB‐122) cells were purchased from American Type Culture Collection (ATCC, Manassas, VA, USA) and cultured in Dulbecco's modified Eagle's medium (DMEM; #11965; Gibco, Thermo Fisher Scientific, Waltham, MA, USA) (Hs578T) or RPMI 1640 medium (#11875; Gibco, Thermo Fisher Scientific) (BT‐549) supplemented with 10% FBS (#SH30910.03; Thermo Fisher Scientific or #10270‐106; Gibco, Thermo Fisher Scientific), 50 U·mL^−1^ penicillin, 50 μg·mL^−1^ streptomycin (penicillin–streptomycin; #15070‐063; Gibco, Thermo Fisher Scientific), and 0.01 mg·mL^−1^ (Hs578T) or 0.00095 mg·mL^−1^ (BT‐549) of insulin (#12585‐014; Gibco, Thermo Fisher Scientific). The 4T1 cells were also obtained from ATCC and the THP‐1 cells were from JCRB Cell Bank (Osaka, Japan). Both the 4T1 and THP‐1 cells were cultured in RPMI 1640 media supplemented with 10% FBS, 50 U·mL^−1^ penicillin, and 50 μg·mL^−1^ streptomycin. Lenti‐X 293T cells were obtained from Clontech Laboratories (Takara Bio, Shiga, Japan) and cultured in DMEM supplemented with 10% FBS, 50 U·mL^−1^ penicillin, 50 μg·mL^−1^ streptomycin, 2 mm‐Glutamine (#25030‐081; Gibco, Thermo Fisher Scientific), 1 × MEM‐NEAA (#11140‐050; Gibco, Thermo Fisher Scientific), and 1 mm sodium pyruvate (#11360‐070; Gibco, Thermo Fisher Scientific). Cells were maintained in a 5% CO_2_ atmosphere at 37 °C.

### 2.2 Reagents

Recombinant human TGF‐β (TGF‐β3; #243‐B3‐010) was obtained from R&D Systems (Minneapolis, MN, USA). Nigericin (#tlrl‐nig) was purchased from InvivoGen (San Diego, CA, USA), and lipopolysaccharide (LPS) (#L4391) was from Sigma‐Aldrich (Merck, Darmstadt, Germany). Z‐VAD‐FMK (#G7232) was from Promega (Madison, WI, USA), and Z‐DEVD‐FMK (#FMK004) was from R&D Systems. SB202190 (#ab120638) was from Abcam (Cambridge, UK), and SB203580 (#tlrl‐sb20) was from InvivoGen. LY364947 was from Calbiochem (#616451; Merck).

### 2.3 PolyI:C transfection and RNA interference

PolyI:C (#tlrl‐pic) was purchased from InvivoGen. PolyI:C transfection was performed using Lipofectamine 2000 Transfection Reagent (#11668‐019; Invitrogen, Thermo Fisher Scientific) as recommended by the manufacturer’s protocol. Transfection of small interfering RNAs (siRNAs) was performed using Lipofectamine RNAiMAX Transfection Reagent (#13778‐150, Invitrogen, Thermo Fisher Scientific) according to the manufacturer’s protocol. We used the following Stealth RNAi siRNAs from Thermo Fisher Scientific: siGSDMD, GGGACAACGUGUACGUGGUGACUGA and UCAGUCACCACGUACACGUUGUCCC; siGSDME, CCAGGCGGUCCUAUUUGAUGAUGAA and UUCAUCAUCAAAUAGGACCGCCUGG. For the transfection of control siRNA, Stealth RNAi siRNA Negative Control Hi GC Duplex #2 (#12935114, Invitrogen, Thermo Fisher Scientific) was used. The final concentration of siRNA in the culture medium was 50 nm. Cells were lysed 60 h post‐transfection of siRNAs.

### 2.4 RNA extraction and quantitative reverse transcription–polymerase chain reaction (qRT‐PCR)

Total RNA was extracted using an RNeasy Mini Kit (#74106; QIAGEN, Hilden, Germany) in accordance with the manufacturer's protocol. First‐strand cDNAs were synthesized using a PrimeScript II 1st strand cDNA Synthesis Kit (#6210A; Takara Bio). The qRT‐PCR was performed using FastStart Universal SYBR Green Master (Rox) (#04913914001; Roche Diagnostics, Basel, Switzerland) with a StepOnePlus Real‐Time PCR System (#4376357; Thermo Fisher Scientific). PCR data were normalized to the expression of *TBP*. Primer sequences are provided in Table S1.

### 2.5 RNA sequence (RNA‐seq) analysis

To prepare libraries from the cells for RNA‐seq, an RNeasy Mini Kit with RNase‐Free DNase Set (QIAGEN), Dynabeads mRNA DIRECT Micro Purification Kit, and Ion Total RNA‐Seq Kit v2 (Thermo Fisher Scientific) were used according to the manufacturers' protocols. cDNA libraries were quantified using an Ion Library TaqMan Quantitation Kit (Thermo Fisher Scientific). Sequencing was performed with an Ion Proton System (Thermo Fisher Scientific) using an Ion PI Hi‐Q Chef Kit (Thermo Fisher Scientific) and Ion PI Chip Kit v3 (A26770, Thermo Fisher Scientific). Reads were aligned to the human genome (Genome Reference Consortium Human Build 37 (GRCh37)/hg19) as a reference genome using TopHat2 open‐source bioinformatics tool. Fragments per kilobase of exon per million sequence reads (FPKM) values were calculated using the Cuffdiff function of Cufflinks. Raw sequence data have been deposited in the Gene Expression Omnibus (GEO) database (accession number GSE152414).

### 2.6 Gene set enrichment analysis (GSEA)

Mean FPKM values were calculated from duplicate biological samples for each condition. Genes used for GSEA were extracted using the threshold value of ≥ 10 FPKM for any of the conditions. Analysis was performed using default parameters. The chip platform ‘Human_NCBI_Entrez_Gene_ID_MSigDB.v7.1.chip’ was used as gene symbol data. Gene ontology gene sets ‘M_UP’ and ‘IM_DOWN’ were obtained from Lehmann *et al*. [25].

### 2.7 Gene Ontology analysis

The reads per kilobase of exon per million sequence reads (RPKM) value of each gene in the cancer cell lines was obtained from Broad Institute Cancer Cell Line Encyclopedia (CCLE, ‘CCLE_DepMap_18q3_RNAseq_RPKM_20180718.gct’). Gene ontology of each gene list was analyzed using the analysis tool ‘Enrichr’ (https://amp.pharm.mssm.edu/Enrichr/) [26, 27].

### 2.8 Immunoblotting

After washing with PBS, cells were lysed with RIPA buffer (50 mm Tris/HCl, pH 8.0; 150 mm NaCl; 1% Triton X‐100; 0.1% SDS; and 0.5% sodium deoxycholate) containing cOmplete EDTA‐free Protease Inhibitor Cocktail Tablets (#05056489001; Roche Diagnostics) and Phosphatase Inhibitor Cocktail (EDTA‐free; #07575‐51; Nacalai Tesque, Kyoto, Japan). To detect gasdermin D (GSDMD) and gasdermin E (GSDME), lysed samples were sonicated twice at power high for 30 s each time at intervals of 30 s using a Bioruptor UCD‐200™ sonicator (Cosmo Bio, Tokyo, Japan). Cell debris was removed by centrifugation (20 400 ***g***, 10 min, 4 °C). Protein amounts in all samples were measured and normalized using Pierce bicinchoninic acid Protein Assay Reagent A/B (#23223, #23224; Thermo Fisher Scientific). SDS/PAGE was performed to electrophoretically separate the proteins based on size, and the samples were then transferred to Fluoro Trans W membranes (#BSP0161; Pall Corporation, Port Washington, NY, USA). The membranes were blocked by 5% skim milk in Tris‐buffered saline with Tween‐20 (TBS‐T). After washing with TBS‐T, the membranes were incubated with antibodies diluted in Can Get Signal Solution 1 (#NKB‐201; TOYOBO, Osaka, Japan). Blots were visualized using a LAS‐4000 image analysis system (Fujifilm, Tokyo, Japan).

The following antibodies were used: anti‐GSDME (#ab215191), anti‐Smad3 (#ab40854), anti‐phospho‐Smad3 Ser423/Ser425 (#ab52903), and anti‐phospho‐IRF3 Ser386 (#ab76493) antibodies purchased from Abcam; anti‐GSDMD (#93709), anti‐phospho‐IRF3 Ser396 (#4947), anti‐MDA5 (#5321), anti‐RIG‐I (#3743), anti‐Noxa (#14766), anti‐phospho‐p38 MAPK Thr180/Tyr182 (#9211), anti‐p38α MAPK (#9228), and anti‐HSP27 (#2402) antibodies from Cell Signaling Technology (Danvers, MA, USA); anti‐GAPDH (#G8795) and anti‐phospho‐HSP27 Ser78 (#05‐645) antibodies from Sigma‐Aldrich (Merck); and anti‐IRF3 antibody (Cat #655704) from BioLegend (San Diego, CA, USA).

### 2.9 Annexin V and PI staining

After collecting all cells, including both the floating cells and attached cells, the dying or dead cells were detected using an eBioscience Annexin V Apoptosis Detection Kit APC (#88‐8007‐72; Thermo Fisher Scientific) according to the manufacturer's protocol. Flow cytometry was performed using a Gallios Flow Cytometer (Beckman Coulter, Brea, CA, USA), and the data were analyzed using flowjo software (BD, Franklin Lakes, NJ, USA).

### 2.10 Microscopy

To evaluate the cell state after polyI:C transfection and capture images, an IX70 microscope with DP72 microscope digital camera (Olympus, Tokyo, Japan) or a BZ‐X710 microscope (Keyence, Osaka, Japan) was used.

### 2.11 Lactate dehydrogenase (LDH) release assay

To determine LDH values (%) after 60 and 72 h of transfection, Hs578T cells were seeded into 96‐well plates at a density of 2 × 10^4^ cells/well. To determine LDH values (%) after 36 and 48 h of transfection or of mock transfections, Hs578T cells were seeded into 96‐well plates at a density of 1 × 10^4^ cells/well to prevent the cells from being confluent. The % LDH values were calculated using a CytoTox 96 Non‐Radioactive Cytotoxicity Assay (#G1780; Promega) according to the manufacturer's protocol. After 10 min of reaction time, stop solution was added and absorbance at 490 nm was measured using a Model 680 Microplate Reader (Bio‐Rad, Hercules, CA, USA).

### 2.12 Measurement of caspase 3/7 activity

Hs578T cells expressing HA or constitutively active Smad3 (caSmad3) were seeded into 96‐well plates at a density of 1 × 10^4^ cells/well. A Caspase‐Glo 3/7 Assay System (#G8090; Promega) was used to measure the activity of caspase 3/7 according to the manufacturer's protocol by using one plate. After 1 h of substrate incubation, luminescent signals were measured using a Mithras LB 940FP Microplate Reader (Berthold Technologies, Bad Wildbad, Germany). Another plate was used to count viable cells using trypan blue staining.

### 2.13 Chromatin immunoprecipitation (ChIP) sequence data of Smad2

Anti‐Smad2 ChIP sequence data from Hs578T cells stimulated with activin A were obtained in our previous study (GEO, accession number GSM3301952) [28]. Smad2 binding at the target gene locus was shown using the UCSC Genome Browser on Human Dec. 2013 (GRCh38/hg38) Assembly.

### 2.14 Lentivirus production and infection

A plasmid lentiCRISPR v2 was a gift from Feng Zhang (#52961; Addgene, Watertown, MA, USA; http://n2t.net/addgene:52961). For constructing lentiviral vectors containing Cas9 and guide RNAs (gRNAs), gene‐specific gRNA was designed using CHOPCHOP (https://chopchop.cbu.uib.no/) and inserted into lentiCRISPR v2. The gRNA sequences for each gene or control gRNA [29] are described in Table S2. caSmad3 cDNA and CSII‐CAG‐MCS‐IRES‐puro lentiviral vector were described previously [28]. Briefly, caSmad3 or HA control tag was subcloned into the multiple cloning site (MCS) of CSII‐CAG‐MCS‐IRES‐puro lentiviral vector, which is originated from CSII‐EF‐RfA to express the inserted cDNA driven by CAG promoter. CSII‐EF‐RfA was obtained from late H. Miyoshi (formerly Keio University, Tokyo, Japan). For caSmad3 cDNA, the C‐terminal phosphorylation site of Smad3 (SSVS) was changed to DDVD [30]. For production of lentivirus, the above plasmids were cotransfected with pCMV‐VSV‐G‐RSV‐Rev and pCAG‐HIVgp (from late H. Miyoshi) into Lenti‐X‐293T cells. Culture media containing lentivirus were filtered through a 0.45‐μm filter and concentrated using a Lenti‐X Concentrator (#631232; Takara Bio). Infected cells were treated with puromycin (ant‐pr; InvivoGen) for 4 days (Hs578T cells, 10 μg·mL^−1^; 4T1 cells, 10 μg·mL^−1^) or 5 days (BT‐549 cells, 10 μg·mL^−1^). Hs578T cells stably expressing caSmad3 were used for analysis within 1 month of culture.

### 2.15 *In vivo* polyI:C treatment

*In vivo* experiments were performed according to the policies of the Animal Ethics Committee of the University of Tokyo (approval number: Med.‐P16‐140). Mice were obtained from Japan SLC Inc. (Shizuoka, Japan). A total of 5 × 10^5^ 4T1 cells were inoculated into the mammary fat pad of 8‐ to 10‐week‐old BALB/c female mice that were anesthetized with isoflurane (#099‐06571; FUJIFILM Wako Pure Chemical Corporation, Osaka, Japan). Tumor sizes were measured using a caliper, and tumor volumes (V) were calculated using the formula: V = (length) × (width)^2^/2.

Intratumoral polyI:C delivery was performed using *in vivo* jetPEI transfection reagent with glucose solution (#201; Polyplus‐transfection, Illkirch, France) in accordance with the manufacturer's protocol with an ionic balance of N/P = 6. A total volume of 100 μL reagent containing polyI:C (20 μg/mouse) or an equivalent amount of distilled water was intratumorally injected into the tumors of mice that were anesthetized with isoflurane. Tumor weight was measured at day 21 or at the day of endpoint [weight loss (> 20% or > 10%/week), mean diameter > 1.2 cm, or general appearance]. Tumor samples were snap‐frozen and stored at −80 °C until use.

### 2.16 Immunohistochemistry

Tumor samples were sectioned with 7 μm thickness and fixed in 4% paraformaldehyde for 10 min. The tumor sections were washed and then blocked with Blocking One reagent (#03953‐95; Nacalai Tesque). The blocked specimens were incubated with primary antibodies in Blocking One reagent at 4 °C overnight. After washing the plates, the samples were incubated with secondary antibody in Blocking One reagent at 37 °C for 30 min. Washed plates were mounted with 4′,6‐diamidino‐2‐phenylindole (DAPI) Fluoromount‐G (#0100‐20; SouthernBiotech, Birmingham, AL, USA) and analyzed using a BZ‐X710 microscope (Keyence). Nonspecific signals with strong intensity were removed. The following antibodies were used: SMAD3 phospho‐S423/phospho‐S425 antibody (#600‐401‐919; Rockland Immunochemicals, Limerick, PA, USA), rabbit IgG‐UNLB (isotype control, #0111‐01; SouthernBiotech), and goat anti‐rabbit IgG (H + L) cross‐adsorbed secondary antibody, Alexa Fluor 488 (#A‐11008; Invitrogen, Thermo Fisher Scientific).

### 2.17 Statistical analysis

Student's *t*‐test was used for comparison between two samples, and the Tukey–Kramer test was used for comparison of multiple samples.

## 3 Results

### 3.1 TGF‐β signaling was suppressed by transfection of polyI:C

**Fig. 1:**
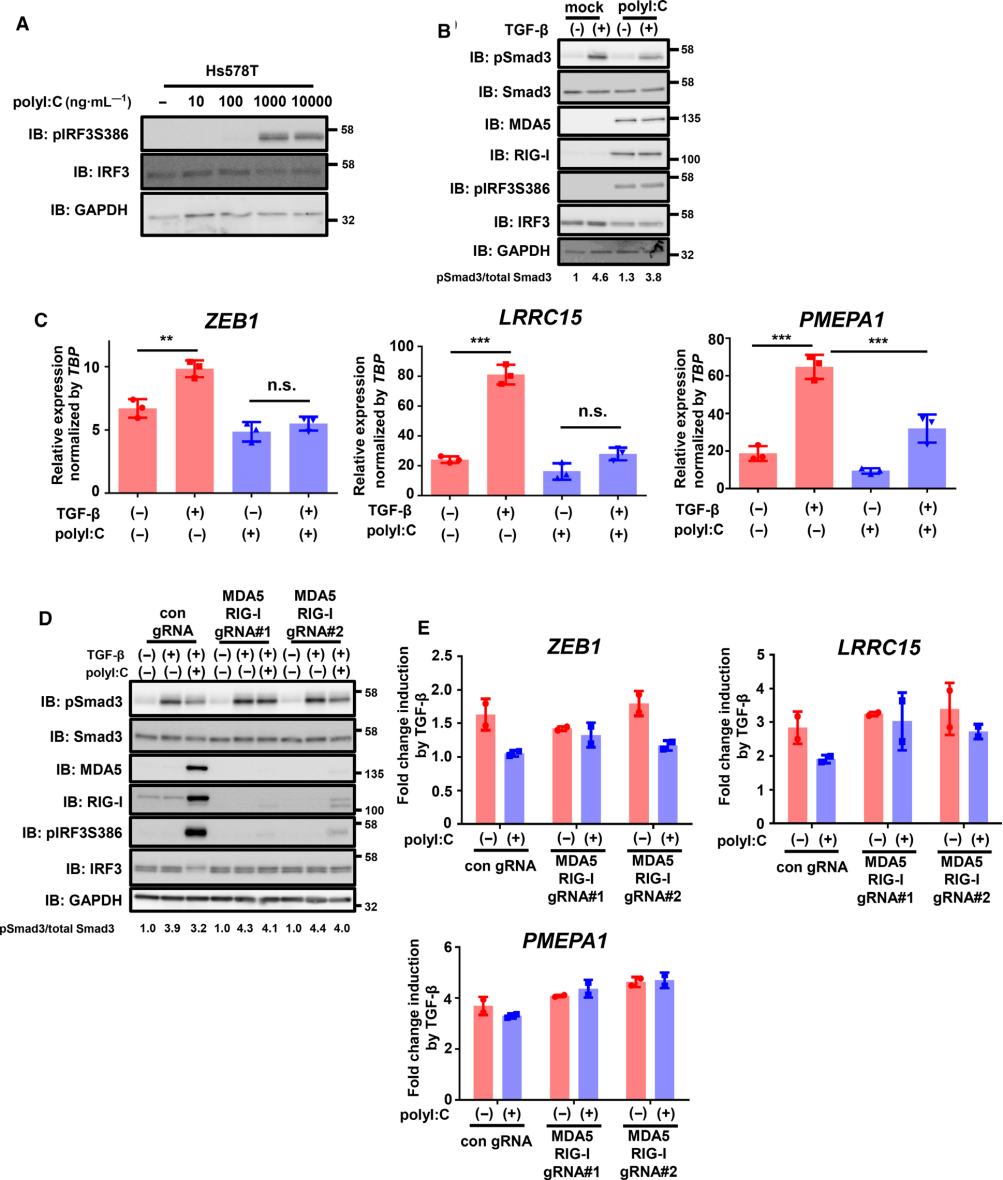
PolyI:C inhibits TGF‐β signaling via RIG‐I‐like receptors. (A) Immunoblotting for phosphorylated IRF3 after 7.5 h of transfection of polyI:C in Hs578T cells. IB: immunoblotting. (B) Effect of polyI:C (1 μg·mL^−1^) on the activation of Smad3 by TGF‐β in Hs578T cells. After 12 h of polyI:C transfection, cells were stimulated with TGF‐β (1 ng·mL^−1^) for 1.5 h and then harvested. Representative data from the two independent experiments are shown. (C) qRT‐PCR analysis of *ZEB1, LRRC15*, and *PMEPA1* expression in Hs578T cells after stimulation with TGF‐β with or without transfection of polyI:C. After 12 h of polyI:C transfection (1 μg·mL^−1^), cells were stimulated with TGF‐β (1 ng·mL^−1^) for 6 h. Expression levels were normalized to *TBP* expression. Data are shown as the mean of the three biological replicate samples. Error bars indicate the SD. **P < 0.01, ***P < 0.001, n.s.: not significant by the Tukey–Kramer test. (D, E) Effect of gene inactivation of MDA5 and RIG‐I on polyI:C inhibition of TGF‐β signaling. Hs578T‐Cas9 cells constitutively expressing gRNAs targeting MDA5 and RIG‐I genes or nontarget control gRNA were used for analysis. (D) After 12 h of polyI:C transfection (1 μg·mL^−1^), cells were stimulated with TGF‐β (1 ng·mL^−1^) for 1.5 h. Immunoblotting for MDA5 and RIG‐I shows the gene inactivation efficiency. Activation of TGF‐β signaling was assessed by immunoblotting of phosphorylated Smad3. Representative data of the three independent experiments are shown. (E) qRT‐PCR analysis of *ZEB1, LRRC15*, and *PMEPA1* expression in Hs578T‐Cas9 cells after stimulation with TGF‐β with or without transfection of polyI:C. After 12 h of polyI:C transfection (1 μg·mL^−1^), cells were stimulated with TGF‐β (1 ng·mL^−1^) for 6 h. Data are shown as the mean of two biological replicates. Error bars indicate the SD.

To evaluate whether the activation of RLR signaling induced by the transfection of polyI:C regulates TGF‐β signaling, we first confirmed the activation of RLR signaling in TNBC cells. We chose the widely used TNBC cell line Hs578T in which the TGF‐β signaling pathway is retained and the phosphorylation of Smad3 by TGF‐β is reported to occur [31]. Strong C‐terminal phosphorylation of IRF3 at Ser386, which is critical for its activation [32], was observed following the transfection of 1 μg·mL^−1^ of polyI:C in Hs578T cells (Fig. 1A). As previously reported, the amount of total IRF3 was downregulated by activation of PRR signaling [33, 34]. We then evaluated the effect of polyI:C on TGF‐β signaling and found that polyI:C suppressed the phosphorylation of Smad3 with strong induction of MDA5 and RIG‐I, which is a known positive feedback mechanism (Fig. 1B). We also found that increased expression of the known target genes of TGF‐β, *ZEB1*, *LRRC15* [35], and *PMEPA1* was suppressed by polyI:C (Fig. 1C). Of note, expression of *SERPINE1*, which is also a well‐known target of both TGF‐β and inflammatory response, was markedly increased by polyI:C even in the absence of TGF‐β stimulation, and *SMAD7* is not significantly induced by TGF‐β in this experimental setting (Fig. S1A). The above data suggested that polyI:C attenuated TGF‐β signaling and the expression of its target genes by suppressing the phosphorylation of Smad3.

We next examined whether the attenuation of TGF‐β signaling by polyI:C was dependent on RLRs. Gene inactivation of both MDA5 and RIG‐I using lentiviral expression of Cas9 and gRNAs targeting MDA5 and RIG‐I suppressed the polyI:C‐induced attenuation of Smad3 phosphorylation, while polyI:C‐induced phosphorylation of IRF3 was strongly reduced by both MDA5 and RIG‐I gRNAs (Fig. 1D). In addition, suppression of TGF‐β‐induced target gene expression by polyI:C was partly attenuated by MDA5 and RIG‐I gRNAs (Fig. 1E). The gene inactivation efficiency of MDA5 and RIG‐I was also confirmed by the downregulation of *IFNB1* expression by MDA5 and RIG‐I gRNAs, which is a common target of activated IRF3. We observed better inactivation efficiency using pair #1 gRNAs compared with that using pair #2 gRNAs (Figs 1D and S1B). These results suggested that polyI:C suppressed TGF‐β signaling in an MDA5‐ and RIG‐I‐dependent manner.

We also evaluated the potential for inhibition of TGF‐β signaling by cell‐intrinsic activation of RLR signaling. Gene ontology analysis of genes extracted from the CCLE database that negatively correlated with MDA5 expression in TNBC cell lines showed that the only enriched ontology was TGF‐β signaling from the dataset ‘Panther_2016’, while the most positively correlated gene set was ‘Toll receptor signaling pathway’ as expected (Fig. S2A). Using another ontology dataset, ‘WikiPathways_2019_Human’, we confirmed that the ontology of TGF‐β signaling was significantly enriched in genes inversely correlated with MDA5 expression in TNBC, while ontologies of positively correlated gene sets were associated with interferon signaling (Fig. S2B). We also performed RNA‐seq analysis of Hs578T cells with or without TGF‐β stimulation to determine the global target of TGF‐β signaling in these TNBC cells (Table S3). Using the RNA‐seq data, GSEA was performed for the genes that inversely correlated with MDA5. We found that many of the genes inversely correlated with MDA5 expression were targets of TGF‐β signaling (Fig. S2C). We also studied the molecular subtype of TNBC [25]. Many upregulated genes in the mesenchymal subtype of TNBC, in which activation of TGF‐β signaling has been reported, were also upregulated by TGF‐β stimulation (Fig. S2D). On the other hand, many downregulated genes in the immunomodulatory subtype that are characterized by the activation of immune cell signaling were also increased by TGF‐β stimulation of Hs578T cells (Fig. S2E). These results suggested the possibility that endogenous activation of RLR signaling can also suppress TGF‐β signaling.

### 3.2 Attenuation of TGF‐β signaling by polyI:C promoted cell death

**Fig. 2:**
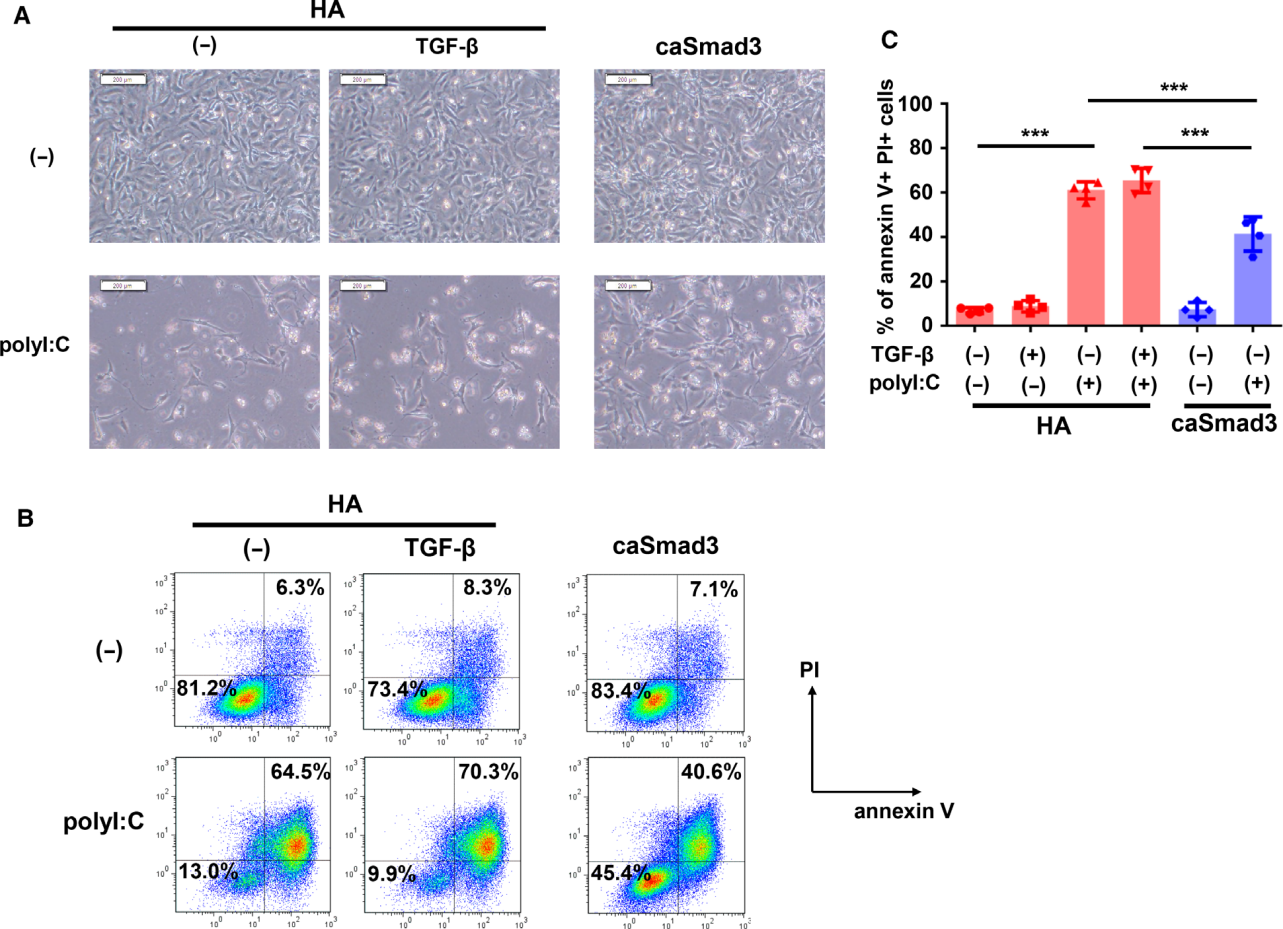
PolyI:C accelerates cell death through the inhibition of TGF‐β signaling. Effect of forced expression of constitutively active Smad3 (caSmad3) on polyI:C‐induced cell death. Hs578T cells expressing HA (control) or caSmad3 were transfected with polyI:C (1 μg·mL^−1^). Twelve hours after transfection, cells were starved with serum‐free culture media and then stimulated with TGF‐β (1 ng·mL^−1^) or left untreated. After 36 h of starvation, the cells were analyzed with the following experiment. (A) Phase‐contrast micrographic images of the Hs578T cells demonstrating the effect of polyI:C transfection and expression of caSmad3. Scale bars, 200 μm. (B, C) Flow cytometric analysis of polyI:C‐transfected Hs578T cells stained with annexin V‐APC and PI. The percentage of annexin V‐positive and PI‐positive cells in forward scatter (FSC)‐gated and side scatter (SSC)‐gated cells are shown. Representative data (B) and mean values (C) of four biological replicate samples are shown. Error bars indicate the SD. ***P < 0.001 by the Tukey–Kramer test.

Because the induction of cell death is one of the marked phenotypes of polyI:C transfection [7, 11], we examined whether the attenuation of TGF‐β signaling by polyI:C would accelerate cell death in Hs578T cells. To investigate the biological effect of polyI:C‐induced suppression of TGF‐β signaling, we established Hs578T cells stably expressing caSmad3 in which the C‐terminal phosphorylation site of Smad3 was changed from SSVS to DDVD [30] (Fig. S3A). Exogenous expression of caSmad3 was expected to activate the signaling pathway at a point downstream of signal inhibition by polyI:C. PolyI:C transfection induced the damaged floating cells in the culture, while the stable expression of caSmad3 markedly counteracted the effect of polyI:C and increased the intact cells attached to the plate (Fig. 2A). We then evaluated polyI:C‐induced cell death using flow cytometry and staining with annexin V and propidium iodide (PI). We found that the expression of caSmad3 decreased the proportions of annexin V^+^/PI^+^ cells, which were remarkably increased by polyI:C transfection (Fig. 2B,C). Of note, stimulation with TGF‐β did not halt cell death possibly because the amount of phosphorylated Smad3 was suppressed by polyI:C and not enough for protection from cell death. We additionally stimulated Hs578T cells with TGF‐β before polyI:C transfection, and found that pretreatment with TGF‐β very weakly suppressed polyI:C‐induced cell death, although the effect of TGF‐β was not significant (Fig. S3B). These results suggested that the activation of the downstream signaling pathway by expression of caSmad3 in Hs578T cells partly rescued the cells from polyI:C‐induced cell death.

### 3.3 Characterization of polyI:C‐induced cell death

**Fig. 3:**
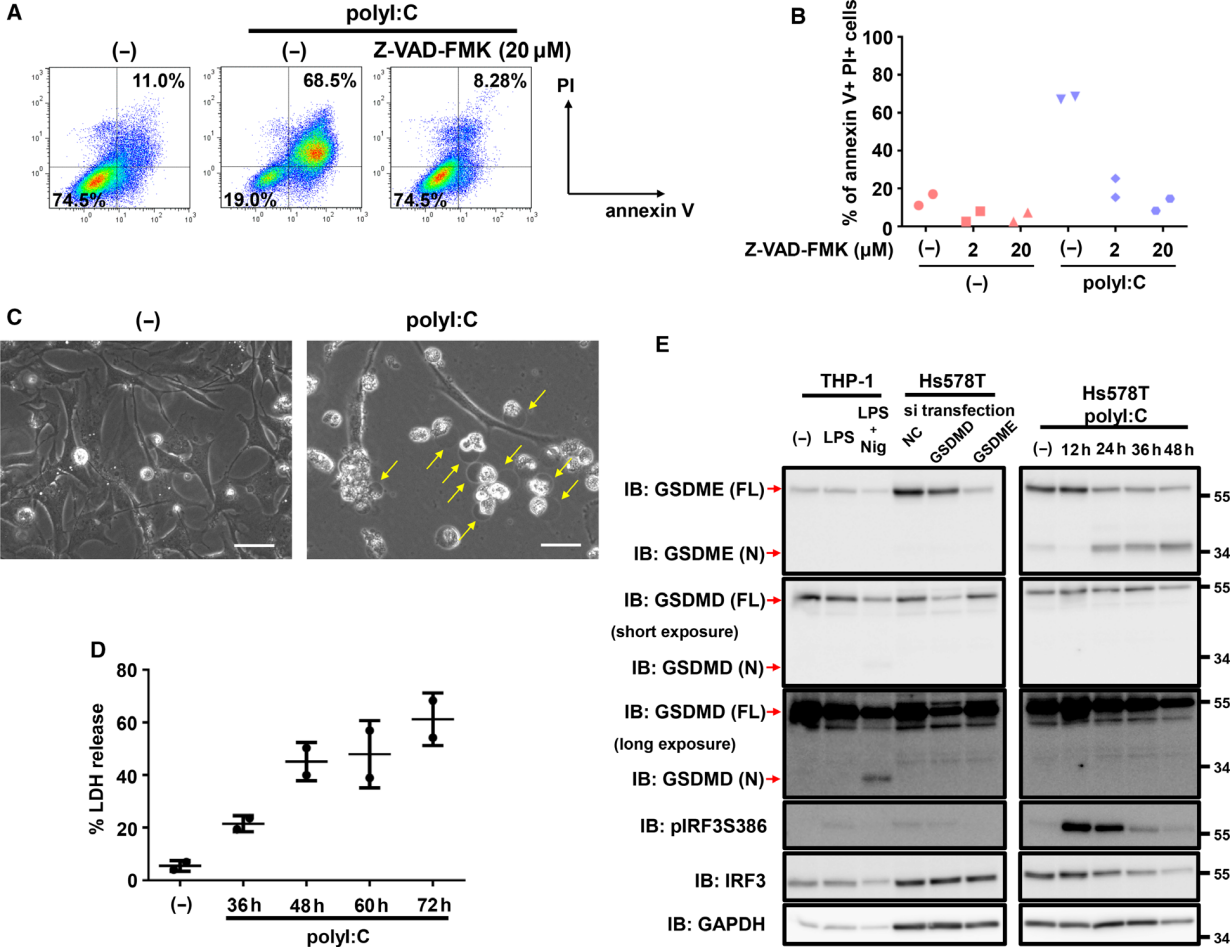
Characteristics of polyI:C‐induced cell death. (A, B) Flow cytometric analysis of polyI:C‐transfected cells with or without Z‐VAD‐FMK treatment. Prior to polyI:C transfection, Hs578T‐HA cells were treated with Z‐VAD‐FMK. Twelve hours after transfection, culture media were changed to serum‐free media with or without Z‐VAD‐FMK. Cells were collected after 36 h of serum starvation and stained with annexin V‐APC and PI. The percentage of annexin V‐positive and PI‐positive populations in the FSC‐gated and SSC‐gated cells are shown. Data were obtained from two biological replicate samples in panel (B). (C) Phase‐contrast micrographic imaging of polyI:C‐transfected Hs578T‐HA cells. Arrows indicate swelling cells. Twelve hours after transfection, culture media were changed to serum‐free media. Cells were analyzed after 36 h of serum starvation. Scale bars, 50 μm. (D) Percentage of LDH released from Hs578T cells transfected with polyI:C (1 μg·mL^−1^) at the indicated time points post‐transfection. Data are shown as the mean of two biological replicate samples. Error bars indicate the SD. (E) Immunoblotting for GSDME and GSDMD after transfection of polyI:C (1 μg·mL^−1^) in Hs578T cells for the indicated time. Twelve hours after transfection, culture media were changed to serum‐free media. Identities of the GSDMD and GSDME bands were confirmed using GSDMD‐knocked down and GSDME‐knocked down Hs578T cells and THP1 cells primed by LPS (10 μg·mL^−1^, 48 h) and treated with nigericin (20 μm, 2 h). Representative data from two independent experiments are shown.

To understand the therapeutic efficacy of polyI:C transfection on TNBC, we further characterized the polyI:C‐induced cell death. As caspase dependency is an important factor for characterizing cell death, we treated polyI:C‐transfected Hs578T cells with the pan‐caspase inhibitor Z‐VAD‐FMK. We found that Z‐VAD‐FMK treatment dramatically decreased the proportion of annexin V^+^/PI^+^ cells that were markedly increased by polyI:C, suggesting that polyI:C‐induced cell death was largely dependent on caspase function (Fig. 3A,B). A previous report suggested that RLR‐induced caspase‐dependent cell death is mediated by intrinsic apoptosis, extrinsic apoptosis, and pyroptosis [11]. Although the definition of ‘pyroptosis’ has changed with time, it is currently believed that pyroptosis is a kind of programmed necrotic cell death induced by gasdermins [36, 37]. Therefore, we examined whether the polyI:C‐induced cell death was pyroptosis. After transfection of Hs578T cells with polyI:C, we observed cell swelling with large bubbles (Fig. 3C), the release of cellular contents quantified by LDH (Fig. 3D), and the absence of annexin V^+^ and PI^‐^ early apoptotic population (Figs. 2B and 3A), which are important phenotypes of pyroptosis. In addition, we evaluated gasdermin cleavage by blotting the N terminus of GSDMD and GSDME. GSDMD and GSDME of the gasdermin family are known to be endogenously cleaved and contribute to pyroptosis. While we did not detect GSDMD cleavage after polyI:C transfection of Hs578T cells, cleavage of GSDMD was detected in THP‐1 cells following the combination of LPS and nigericin, which was used as a positive control (Fig. 3E). In contrast, N‐terminal‐cleaved GSDME was detected in polyI:C‐transfected Hs578T cells (Fig. 3E). These results suggested that polyI:C transfection induced pyroptosis in Hs578T cells.

### 3.4 Attenuation of TGF‐β signaling by polyI:C transfection accelerated caspase 3‐dependent pyroptosis

**Fig. 4:**
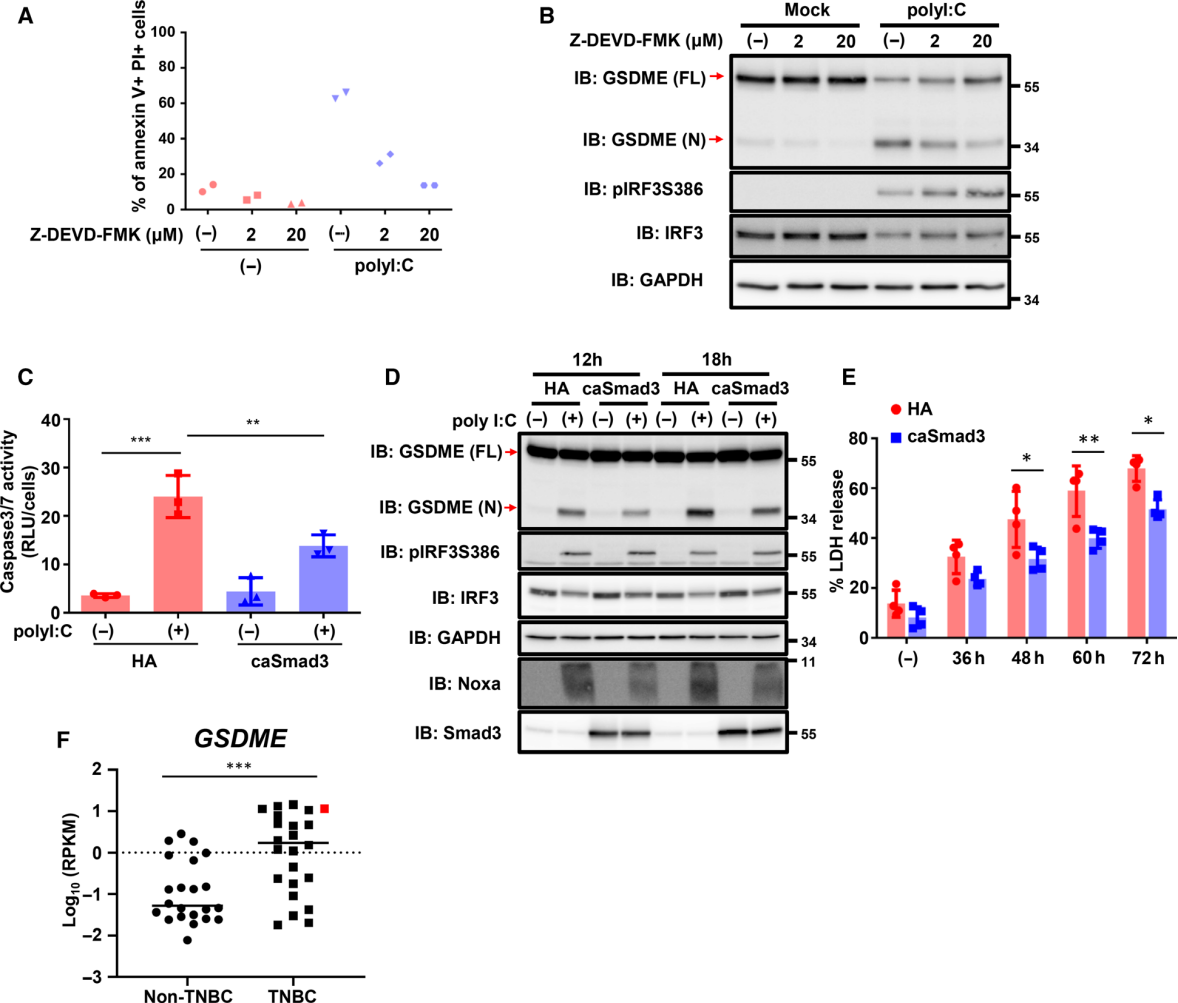
Caspase 3‐dependent pyroptosis induced by polyI:C transfection and the involvement of TGF‐β signaling. (A, B) Prior to transfection of polyI:C (1 μg·mL^−1^) in Hs578T‐HA cells, Z‐DEVD‐FMK was added to the cells. Twelve hours after transfection, culture media were changed to serum‐free media with or without Z‐DEVD‐FMK. (A) Flow cytometry analysis with annexin V‐APC and PI staining was performed 36 h after serum starvation. (B) Cells were lysed and analyzed by immunoblotting for GSDME 12 h postserum starvation. Data were obtained from two biological replicate samples and representative data are shown. (C) Caspase 3/7 activity in Hs578T‐HA or Hs578T‐caSmad3 cells after transfection with polyI:C was assessed using a Caspase‐Glo 3/7 Assay System. Twelve hours after transfection, culture media were changed to serum‐free media. The assay was performed 12 h postserum starvation. Caspase 3/7 activity was normalized to the number of cells that survived. Data are shown as the mean of three biological replicate samples, and the error bars indicate the SD. **P < 0.01, ***P < 0.001 by the Tukey–Kramer test. (D) Immunoblotting of Hs578T‐HA or Hs578T‐caSmad3 cells for GSDME after transfection with polyI:C (1 μg·mL^−1^) at the indicated time points. Representative data from three independent experiments are shown. (E) Percentage of LDH released from Hs578T‐HA or Hs578T‐caSmad3 cells after transfection with polyI:C (1 μg·mL^−1^) at the indicated time points. Data are shown as the mean of four biological replicate samples. Error bars, SD. *P < 0.05, **P < 0.01 by the Tukey–Kramer test. (F) GSDME expression (non‐TNBC cell lines, *n* = 22; vs TNBC cell lines, *n* = 24) deposited in the CCLE database. The red square corresponds to the Hs578T cells. ***P < 0.001 by Student's *t*‐test.

Inhibition of cell death by Z‐VAD‐FMK and detection of GSDME cleavage suggested that activation of the cell death pathway by polyI:C in Hs578T cells was predominantly executed by caspase 3, which is the only known caspase to process and activate GSDME. Therefore, we first used the caspase 3‐specific inhibitor Z‐DEVD‐FMK and found that polyI:C‐induced cell death was almost entirely prevented by Z‐DEVD‐FMK treatment (Fig. 4A). Z‐DEVD‐FMK treatment also inhibited GSDME cleavage induced by polyI:C in Hs578T cells (Fig. 4B). These results suggested that polyI:C‐induced pyroptosis in Hs578T cells was largely dependent on caspase 3 activation.

As described above, attenuation of TGF‐β signaling by polyI:C accelerated Hs578T cell death. Furthermore, the forced expression of caSmad3 in Hs578T cells decreased the proportion of annexin V^+^/PI^+^ cells and directly increased the number of annexin V^‐^/PI^‐^ cells without the appearance of early apoptotic annexin V^+^/PI^‐^ cells. Therefore, the next question we asked was whether caSmad3 expression would attenuate polyI:C‐induced pyroptosis in Hs578T cells. To address this, we measured caspase 3/7 activity and found that caSmad3 expression suppressed caspase 3/7 activation, which was elevated by polyI:C transfection (Fig. 4C). In addition, caSmad3 expression also abrogated polyI:C‐induced GSDME cleavage and the release of LDH (Fig. 4D,E). Expression of GSDME is important for switching from apoptosis to pyroptosis [37]. Although expression of GSDME is lower in all types of breast cancer than that in other types of cancer cells (Fig. S4), we found that GSDME expression in TNBC was significantly higher than that in non‐TNBC [25, 38] (Fig. 4F), supporting a previous report [39]. The above results suggested that TNBC‐specific GSDME expression appeared to contribute to polyI:C‐induced caspase 3‐mediated pyroptosis, which was inhibited by caSmad3 expression.

To examine whether suppression of TGF‐β signaling by polyI:C and its pyroptosis‐promoting effect are universally applicable in TNBC, we additionally used another TNBC cell line, BT‐549, in which TGF‐β also shows tumor‐promoting effects [22]. Transfection of polyI:C suppressed phosphorylation of Smad3 (Fig. S5A) and the induction of TGF‐β target genes *PMEPA1*, *SERPINE1*, and *SMAD7*. We have not observed significant induction of *ZEB1* and *LRRC15* (Fig. S5B) that we have seen in Hs578T cells (Fig. 1C), possibly reflecting the context dependency of the TGF‐β targets [40]. We also checked caspase‐dependent cell death using Z‐VAD‐FMK. PolyI:C transfection notably induced annexin V^+^/PI^+^ populations, and Z‐VAD‐FMK treatment dramatically suppressed these populations, suggesting caspase‐dependent cell death by polyI:C was also observed in BT‐549 cells (Fig. S5C). Significant production of N‐terminal cleaved GSDME, not GSDMD, was also observed in BT‐549 cells by polyI:C transfection (Fig. S5D). Thus, we analogously produced BT‐549 cells stably expressing caSmad3, and evaluated polyI:C‐induced cell death. Expression of caSmad3 prominently suppressed cell death (Fig. S5E–G), and TGF‐β pretreatment showed a trend to partially abrogate polyI:C‐induced cell death (Fig. S5H). Cleavage of GSDME was also attenuated by caSmad3 (Fig. S5I).

### 3.5 Detailed mechanism of protection from polyI:C‐induced cell death by caSmad3

**Fig. 5:**
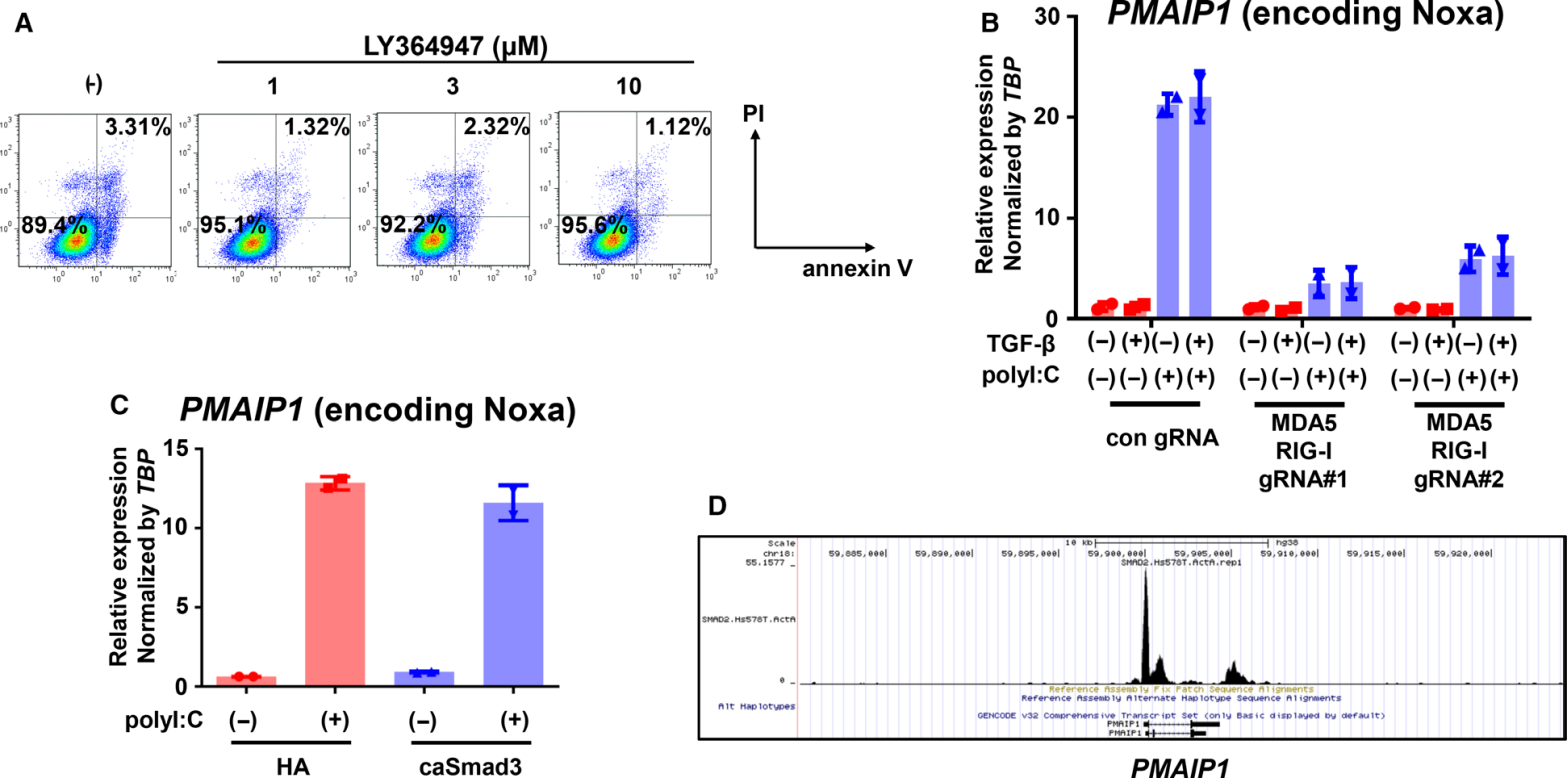
Noxa expression induced by polyI:C is partly inhibited by TGF‐β signaling. (A) Flow cytometric analyses of Hs578T‐HA cells stained with annexin V‐APC and PI were performed 36 h post‐treatment with LY364947, a kinase inhibitor of type I TGF‐β receptor. Data were obtained from cells under serum‐free conditions. (B) qRT‐PCR analysis of Noxa‐encoding *PMAIP1* expression in Hs578T‐Cas9 cells expressing both MDA5 and RIG‐I gRNAs after stimulation with TGF‐β with or without transfection of polyI:C. After 12 h of polyI:C transfection (1 μg·mL^−1^), cells were stimulated with TGF‐β (1 ng·mL^−1^) for 6 h. Data are shown as the mean of two biological replicate samples. Error bars, SD. (C) qRT‐PCR analysis of *PMAIP1* expression in Hs578T‐HA or Hs578T‐caSmad3 cells after 12 h of transfection with polyI:C (1 μg·mL^−1^). Expression levels were normalized by *TBP* expression. Data are shown as the mean of two biological replicate samples. Error bars, SD. (D) Smad2 binding at the *PMAIP1* locus. Smad2 ChIP‐seq data of Hs578T cells treated with activin A (50 ng·mL^−1^) for 1.5 h were used (GEO, accession number GSM3301952).

We then investigated the mechanisms of caSmad3‐triggered cell survival of Hs578T cells following polyI:C transfection. RNA‐seq analysis revealed that TGF‐β stimulation did not dramatically alter antiapoptotic Bcl‐2 factor expression in Hs578T cells (Fig. S6A). Furthermore, treatment with the TβRI inhibitor LY364947 did not increase cell death (Figs. 5A and S6B), suggesting that not the TGF‐β signaling alone but its interference with cell death pathway triggered by polyI:C exhibited cell survival in Hs578T cells. One of the known mechanisms of RLR‐induced cell death is explained by Noxa, a BH3‐only protein. We found that polyI:C dramatically induced Noxa expression, which was suppressed by the gene inactivation of MDA5 and RIG‐I (Fig. 5B). Stimulation of the cells with TGF‐β did not affect the expression of Noxa, which was in accordance with the result observed in the flow cytometry in Fig. 2C. Next, we measured Noxa expression after polyI:C transfection with or without caSmad3 expression. We found that caSmad3 partly suppressed the induction of Noxa mRNA and protein expression (Figs 4D and 5C). Decreased expression of Noxa with caSmad3 expression was also observed in BT‐549 cells (Fig. S5I). To investigate the possibility of transcriptional repression by the Smad pathway downstream of TGF‐β, we reanalyzed the Smad2 ChIP sequence data in Hs578T cells stimulated with the TGF‐β family ligand activin A [28]. We identified a Smad2 binding site in the promoter region of the *PMAIP1* gene encoding Noxa in Hs578T cells (Fig. 5D). A peak in the vicinity of this site was also confirmed in an anti‐Smad2/3 ChIP‐seq data obtained from TGF‐β‐stimulated Hs578T cells, though the peak was weak (data not shown) [19]. These results suggest that Smad‐mediated transcriptional repression may be associated with Noxa downregulation, which then led to the attenuation of polyI:C‐induced cell death by caSmad3 expression.

However, the contribution from inhibition of the Smad pathway to the strong induction of Noxa by polyI:C was limited. Our results therefore suggested that additional mechanisms were involved in the induction of pyroptosis through inhibition of the TGF‐β signaling. Therefore, we examined the association between p38 and the caSmad3‐mediated reduction of cell death. We found that caSmad3 expression attenuated polyI:C‐induced phosphorylation of p38 in Hs578T cells, which could be caused by induction of *DUSP1* by TGF‐β, a known p38 phosphatase (Fig. S7A,B). We used two p38 inhibitors and found that SB203580 partly decreased polyI:C‐induced cell death, but the effect of SB202190 was less (Fig. S7C,D). Together, these results suggested that caSmad3 possibly attenuated polyI:C‐induced pyroptosis through its multiple downstream factors.

### 3.6 Suppression of TGF‐β signaling by polyI:C contributed to tumor suppression

**Fig. 6:**
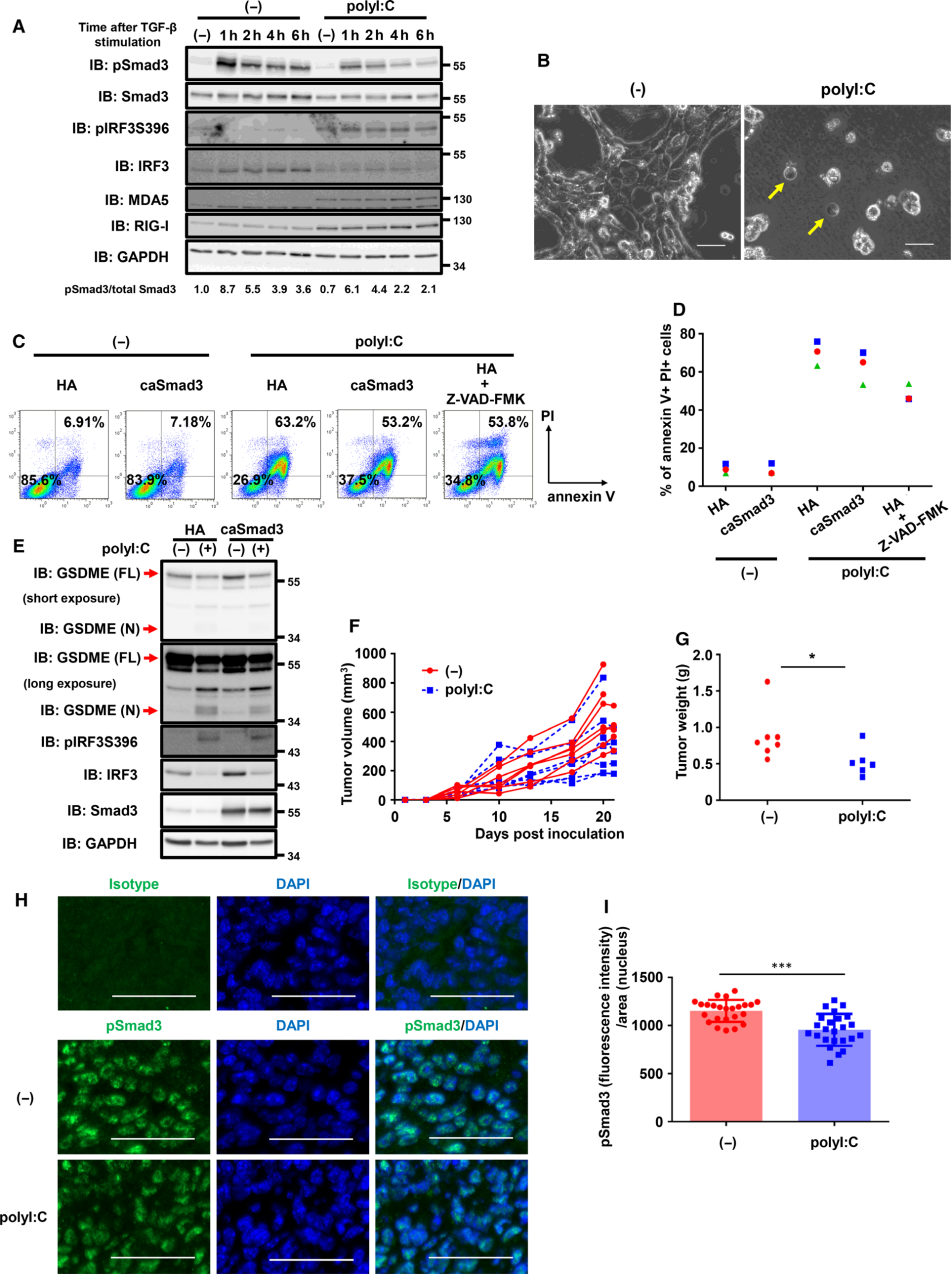
PolyI:C‐induced cell death in 4T1 cells. (A) Immunoblotting for phosphorylated Smad3 in 4T1 cells after polyI:C transfection and TGF‐β stimulation. After 12 h of polyI:C transfection (1 μg·mL^−1^), cells were stimulated with TGF‐β (1 ng·mL^−1^) for the indicated time. Activation of TGF‐β signaling was assessed by immunoblotting of phosphorylated Smad3. Representative data from two independent experiments are shown. (B) Phase‐contrast micrographic imaging of polyI:C‐transfected 4T1‐HA cells. Arrows indicate swelling cells. Twelve hours after polyI:C transfection (1 μg·mL^−1^), culture media were changed to serum‐free media. Cells were analyzed after 36 h of the serum starvation. Scale bar, 50 μm. (C, D) Effect of overexpression of caSmad3 on polyI:C‐induced cell death. 4T1 cells expressing HA (control) or caSmad3 pretreated with or without Z‐VAD‐FMK (50 μm) were transfected with polyI:C (1 μg·mL^−1^). Twelve hours after transfection, culture media were changed to serum‐free media with or without Z‐VAD‐FMK. After 36 h of media change, the cells were analyzed by flow cytometry using annexin V‐APC and PI staining. Percentage of annexin V‐positive and PI‐positive cells in FSC‐gated and SSC‐gated cells are shown. In panel (D), the data obtained from three independent experiments are shown. (E) Immunoblotting for GSDME in 4T1‐HA or 4T1‐caSmad3 cells after 24 h of transfection with polyI:C (1 μg·mL^−1^). Representative data for biologically replicate samples are shown. (F, G) Antitumor effect of polyI:C transfection *in vivo*. BALB/c mice inoculated in the mammary fat pad with 4T1 cells at day 0 were intratumorally transfected with or without polyI:C (20 μg/mouse) on days 10, 13, 17, and 20. Tumor volumes for each mice (F) and tumor weights at the predetermined endpoint or day 21 (G) are shown. Data were obtained from three independent experiments (total of 7 mice for each condition). One mouse injected with polyI:C on day 20 died of unknown reason and was excluded from the evaluation in (G). *P < 0.05 by Student's *t*‐test. (H, I) Immunofluorescence analysis of pSmad3 signals in the nuclei in inoculated 4T1 tumor samples with or without polyI:C treatment. Tumor samples obtained from two mice in panels (F) and (G) were stained using pSmad3 antibody with DAPI. Micrographic images were obtained from 12 microscopic fields for each sample. (H) Representative micrographic images of the isotype control and pSmad3 with or without polyI:C treatment are shown. Scale bar, 50 μm. (I) Fluorescence intensities of nuclear pSmad3 normalized to signal area of the nucleus for each microscopic field were plotted (*n* = 24). Error bars, SD. ***P < 0.001 by Student's *t*‐test.

Finally, we addressed the efficacy of polyI:C suppression of TGF‐β signaling *in vivo*. We used murine breast cancer 4T1 cells, which have been previously used as a model of TNBC [41]. PolyI:C transfection in 4T1 cells attenuated the phosphorylation of Smad3 following TGF‐β stimulation (Fig. 6A). We then evaluated polyI:C‐induced cell death in 4T1 cells. Cell swelling, direct transition of cellular annexin V^‐^/PI^‐^ populations to annexin V^+^/PI^+^ populations, and cleavage of GSDME protein were all observed in 4T1 cells after polyI:C transfection, suggesting that polyI:C also promoted pyroptosis in 4T1 cells (Fig. 6B–E). In addition, we also examined the effect of caSmad3 overexpression on polyI:C‐induced cell death. Forced expression of caSmad3 partially inhibited polyI:C‐induced cell death (Fig. 6C,D) and GSDME cleavage (Fig. 6E). Treatment with Z‐VAD‐FMK also partially suppressed polyI:C‐induced cell death (Fig. 6C,D). These results suggested that both caspase‐dependent and caspase‐independent mechanisms of cell death induced by polyI:C were involved in the 4T1 cells and that this was a critical reason for the partial suppression of polyI:C‐induced cell death by caSmad3. We then evaluated the effect of intratumoral polyI:C transfection on tumor suppression using 4T1 cells orthotopically transplanted into BALB/c mice. Although polyI:C transfection did not significantly affect tumor volume, tumor weight was significantly reduced by polyI:C transfection (Fig. 6F,G) and phosphorylation of Smad3 in tumor samples was significantly reduced after polyI:C transfection (Fig. 6H,I). These results suggested that transfection of polyI:C induced pyroptosis in 4T1 cells, leading to the suppression of tumor growth, which was partially mediated by attenuation of TGF‐β signaling.

## 4 Discussion

The activation of cytosolic dsRNA signaling in cancer cells has been a widely recognized and may be a promising strategy for treating cancers. In the present study, we demonstrated that activation of RLR signaling by transfection of TNBC cells with a synthetic dsRNA analogue suppressed TGF‐β signaling. Furthermore, this process provided additional therapeutic benefit in that attenuation of TGF‐β signaling led to enhancement of cell pyroptosis.

### 4.1 Activation of RLR singling or other PRR signaling in cancer cells

To prevent virus replication, hosts have a system that results in the death of infected cells [42]. It is important to note that this cell death pathway also exists in many cancer cells because many reports have suggested the importance of RLR signaling in tumor suppression. Overexpression of MDA5 [43], endogenous retroviruses [44, 45], cytosolic induction of polyI:C [12, 46, 47, 48], and other ligands recognized by RLR [8] have been reported to increase cell death and tumor repression. Therefore, it is not surprising that polyI:C is being evaluated in a clinical trial of cancer treatment, including TNBC. In the present study, we showed that intracellularly administered polyI:C was recognized by MDA5 and RIG‐I, which resulted in the attenuation of TGF‐β signaling and promotion of cell death. Based on our present findings, other RLR ligands for cancer treatment may show similar inhibitory effects on TGF‐β signaling through the same RLR signaling pathway.

In the context of TNBC treatment, recently approved PARP inhibitors for patients carrying BRCA mutations strikingly inhibit DNA‐repairing system, resulting in breakage of DNA, release of small DNA called ‘micronuclei’ in the cytoplasm, and triggering cyclic GMP‐AMP synthase (cGAS) stimulator of interferon genes (STING) DNA‐sensing pathway that causes activation of TANK‐binding kinase 1 (TBK1) and IRF3 [49, 50]. Therefore, there is a possibility that PARP inhibitor simultaneously suppresses TGF‐β signaling through production of phosphorylated IRF3 by TBK1, and this can be one of the mechanisms of cancer cell death by PARP inhibitor. In addition, activation of nucleic acid sensing pathway such as by cytosolic dsRNA administration or PARP inhibition (with activation of cGAS‐STING signaling) upregulates immune checkpoint proteins [51, 52, 53]. Therefore, the combination therapy of anti‐PD‐1/PD‐L1 antibody with PARP inhibitor or polyI:C is also suggested to suppress TNBC progression partly through the inhibition of TGF‐β pathway. Of note, PARP also negatively regulates TGF‐β‐Smad signaling through ADP‐ribosylation of Smads [54]. Further investigations will be needed regarding the antitumor effect and mode of action of each PARP inhibitor, in relation to its effect on the cGAS‐STING signaling and TGF‐β signaling.

### 4.2 Suppression of TGF‐β signaling by activation of RLR signaling

Signaling interactions sometimes reveal important biological phenotypes. One study showed that knockdown of IRF3 accelerates TGF‐β‐induced EMT in HaCaT normal epidermal keratinocytes, and suggested that activated IRF3 suppresses TGF‐β signaling by binding to Smad3 and blocking its activation by the receptors [24]. This is supported by the findings that the structure of C‐terminal region of IRF3 is similar to that of the MH2 domain of Smad3, which is important for protein–protein interactions [55, 56, 57, 58]. Considering our findings and the fact that TGF‐β is also reported to be important for maintaining cancer stem cells that show resistance to chemotherapeutic drugs in breast cancer, polyI:C transfection may also target caner stem cells.

### 4.3 Prosurvival function of TGF‐β signaling

It is noteworthy that TGF‐β contributes to two contrasting aspects of cell fate. It is reported to exhibit both antisurvival and prosurvival functions, which are dependent on the context and cell types. For example, TGF‐β suppresses antiapoptotic Bcl‐2 and Bcl‐X_L_ expression to induce cell death [59, 60, 61], while in other situations, it induces their expressions to promote cell survival [62, 63, 64]. We focused on the prosurvival function of TGF‐β in this study, which is also observed in microglia, pancreatic β cells, and regulatory T cells by induction of FLICE‐inhibitory protein (FLIP or CFLAR), which suppresses the function of caspase 8 [65, 66, 67]. In the context of cancer, TGF‐β induces *BHLHE40*, which encodes deleted in esophageal cancer 1 (DEC1) to promote survival of mammary carcinoma cells [21]. However, we were unable to detect upregulation of Bcl‐2, Bcl‐X_L_, or FLIP by TGF‐β stimulation (Fig. S6A and data not shown) or the strong induction of DEC1 by caSmad3 expression in Hs578T cells (data not shown). These results indicated that other prosurvival mechanisms of TGF‐β signaling were present in TNBC cells. We suggested that expression of caSmad3 suppressed polyI:C‐induced Noxa expression, which is reported to be important for polyI:C‐induced cell death in cancer cells [46, 48]. This is believed to be mediated through transcriptional suppression by Smads [68]. However, caSmad3‐mediated reduction of Noxa expression is not drastic, leading to the possibility of other systems also being involved in the prosurvival function of TGF‐β in polyI:C‐triggered cell death, such as attenuation of p38 phosphorylation (Fig. S7).

### 4.4 Pyroptosis

The term ‘pyroptosis’ was originally defined as caspase 1‐dependent cell death accompanied by inflammation [69]. However, the definition was changed after the identification of gasdermins as executioners in the permeabilization of plasma membranes [36, 70, 71, 72]. During the late stage of cell death, gasdermins are cleaved by activated caspases or other cytotoxic proteases, releasing the N‐terminal domain, which can trigger pyroptosis by forming oligomers and pores in the cell membrane. This mechanism explains the characteristics of pyroptotic cells we observed. Recent findings suggest that pyroptosis is closely related to cancer progression. Pyroptosis occurs in cancer cells, changing the tumor immune microenvironment from ‘cold’ to ‘hot’ due to the accumulation and activation of antitumor immune cells, such as cytotoxic T cells or natural killer cells. This is believed to be caused by the release of damage‐associated molecular patterns, such as HMGB1, from dead cells [73, 74]. Given that activation of the tumor immune microenvironment is associated with immunotherapy efficacy [75] and GSDME is highly expressed in TNBC [39] (our data), polyI:C‐induced pyroptosis may provide more potent therapeutic efficacy than we expected for the treatment of TNBC. In addition, we showed that caSmad3 expression attenuated polyI:C‐induced pyroptosis through diminishing caspase 3 activity. This may be one reason TGF‐β exhibits immune suppressive activity in the tumor microenvironment.

## 5 Conclusion

Transfection of polyI:C promoted pyroptosis, which was enhanced by the suppression of TGF‐β signaling and its anti‐pyroptotic function. The findings in the present study thus help elucidate a potential molecular targeting strategy associated with the use of synthetic dsRNA analogues for patients with TNBC.

## Conflict of interest

The authors declare no conflict of interest.

## Author contributions

YT, MM, RT, DK, and KM designed the experiment and analyzed the data. YT, DK, and KM wrote the paper. YT performed most of the experiments.

### Peer Review

The peer review history for this article is available at https://publons.com/publon/10.1002/1878‐0261.12890.

## Supporting information


**Fig. S1.** MDA5‐ and RIG‐I‐mediated suppression of TGF‐β signaling.
**Fig. S2.** Potential of cell‐intrinsic activation of RLR signaling and attenuation of TGF‐β signaling.
**Fig. S3.** caSmad3 expression in Hs578T cells and weak inhibition of polyI:C‐induced cell death by the pretreatment of TGF‐β.
**Fig. S4.** *GSDME* expression in various types of cancer cells.
**Fig. S5.** Anti‐pyroptotic effect of TGF‐β is suppressed by polyI:C in BT‐549 cells.
**Fig. S6.** Mechanisms of caSmad3‐mediated cell survival.
**Fig. S7.** Attenuation of p38 phosphorylation by caSmad3 partially inhibits polyI:C‐induced cell death.

**Table S1.** Sequences of the primers.

**Table S2.** Sequences of the gRNAs.

**Table S3.** Top 100 differentially expressed genes by TGF‐β stimulation in Hs578T cells.

## Data Availability

Raw sequence data are available in the GEO database (accession number GSE152414).
