# Supplementary material for: Anti‐pyroptotic function of TGF‐β is suppressed by a synthetic dsRNA analogue in triple negative breast cancer cells
Source: Mol Oncol. 2021 Jan 4;15(5):1289–307. doi: 10.1002/1878-0261.12890 (PMC8096786; doi:10.1002/1878-0261.12890)
Supplement: Supplementary file 1 — Fig. S1. MDA5‐ and RIG‐I‐mediated suppression of TGF‐β signaling. Fig. S2. Potential of cell‐intrinsic activation of RLR signaling and attenuation of TGF‐β signaling. Fig. S3. caSmad3 expression in Hs578T cells and weak inhibition of polyI:C‐induced cell death by the pretreatment of TGF‐β. Fig. S4. GSDME expression in various types of cancer cells. Fig. S5. Anti‐pyroptotic effect of TGF‐β is suppressed by polyI:C in BT‐549 cells. Fig. S6. Mechanisms of caSmad3‐mediated cell survival. Fig. S7. Attenuation of p38 phosphorylation by caSmad3 partially inhibits polyI:C‐induced cell death. [file MOL2-15-1289-s004.zip › mol212890-sup-0011-Legends.docx]

**Anti-pyroptotic function of TGF-β is suppressed by a synthetic dsRNA analogue in triple negative breast cancer cells**

**Supplementary figure legends**

**Figure S1. MDA5- and RIG-I-mediated suppression of TGF-β signaling.**

(A) qRT-PCR analysis of *SERPINE1* and *SMAD7* expression in Hs578T cells after stimulation with TGF-β with or without transfection of polyI:C. After 12 hours of polyI:C transfection (1 μg/mL), cells were stimulated with TGF-β (1 ng/mL) for 6 hours. Samples used in Fig. 1C were used for the analysis. Expression levels were normalized to *TBP* expression. Data are shown as the mean of the three biological replicate samples. Error bars indicate the S.D., n.s.: not significant by Tukey-Kramer test.

(B) qRT-PCR analysis of *IFNB1* expression in Hs578T-Cas9 cells expressing both MDA5 gRNAs and RIG-I gRNAs after stimulation with TGF-β with or without polyI:C. After 12 hours of polyI:C transfection (1 μg/mL), cells were stimulated with TGF-β (1 ng/mL) for 6 hours. Data are shown as the mean of two biological replicates. Error bars, S.D; con gRNA, control gRNA.

**Figure S2. Potential of cell-intrinsic activation of RLR signaling and attenuation of TGF-β signaling**.

(A, B) Gene ontology analysis of positively-correlated and inversely-correlated genes with *IFIH1* (encoding MDA5) expression in TNBC cell lines (n = 24) using the Cancer Cell Line Encyclopedia (CCLE) database. Slope (S) and correlation coefficient (T) of Log_10_ (MDA5 abundance in RPKM + 0.1) + 1 and Log_10_ (gene abundance in RPKM + 0.1) + 1 were calculated and positively-correlated genes (S > 0.2, T^2^ > 0.06, 3,672 genes) or inversely-correlated genes (S < -0.2, T^2^ > 0.06, 1,598 genes) were analyzed using gene ontology datasets “Panther_2016” (A) and “WikiPathways_2019_Human” (B). The top 6 enriched ontologies (p < 0.05) are shown.

(C-E) Gene Set Enrichment Analysis (GSEA) of Hs578T cells stimulated with TGF-β (1 ng/mL) for 24 hours in terms of gene sets inversely correlated with MDA5 used in Supplementary Fig. S2A and S2B (C), upregulated genes in the mesenchymal subtype (M) of TNBC (D), and downregulated genes in the immunomodulatory (IM) subtype (E).

**Figure S3. caSmad3 expression in Hs578T cells and weak inhibition of polyI:C-induced cell death by the pretreatment of TGF-β.**

(A) Immunoblotting for anti-Smad3 in Hs578T cells expressing constitutively active Smad3 (caSmad3).

(B) Effect of TGF-β pretreatment on polyI:C-induced cell death in Hs578T cells. Hs578T cells expressing HA were stimulated with TGF-β (1 ng/mL) for 24 hours before transfection of polyI:C (0.1 or 1 μg/mL). Six hours after transfection, cells were starved with serum-free culture media, and TGF-β stimulation was continued until harvesting. After 48 hours of polyI:C transfection, the cells were analyzed with flow cytometry with annexin V-APC and PI staining. The percentage of annexin V-positive and PI-positive cells in FSC-gated and SSC-gated cells with two biological replicate samples are shown.

**Figure S4. *GSDME* expression in various types of cancer cells.**

The amount of *GSDME* expression in various types of cancer cell lines obtained from the Cancer Cell Line Encyclopedia (CCLE) database. Error bars indicate the S.D.

**Figure S5. Anti-pyroptotic effect of TGF-β is suppressed by polyI:C in BT-549 cells.**

(A) Effect of polyI:C (1 μg/mL) on the activation of Smad3 by TGF-β in BT-549 cells. After 4 hours of polyI:C transfection, cells were stimulated with TGF-β (1 ng/mL) for 1.5 hours and then harvested. Representative data from the three independent experiments are shown.

(B) qRT-PCR analysis of *PMEPA1*, *SERPINE1*, *SMAD7*, *ZEB1*, and *LRRC15* expression in BT-549 cells after stimulation with TGF-β and transfection of polyI:C. After 4 hours of polyI:C transfection (1 μg/mL), cells were stimulated with TGF-β (1 ng/mL) for 6 hours. Expression levels were normalized to *TBP* expression. Data are shown as the mean of the three biological replicate samples. Error bars indicate the S.D. ***P < 0.001, n.s.: not significant by Tukey-Kramer test.

(C) Flow cytometric analysis of polyI:C-transfected cells with or without Z-VAD-FMK treatment. Prior to polyI:C transfection (1 μg/mL), BT-549 cells were treated with Z-VAD-FMK (50 μM). Six hours after polyI:C transfection, culture media were changed to serum-free media with or without Z-VAD-FMK. Cells were collected after 48 hours of polyI:C transfection and stained with annexin V-APC and PI. The percentage of annexin V-positive and PI-positive populations in the FSC-gated and SSC-gated cells are shown. Data were obtained from two biological replicate samples.

(D) Immunoblotting for GSDME and GSDMD after transfection of polyI:C (1 μg/mL) in BT-549 and Hs578T cells. Cells were lysed after 18 hours of transfection. The same sample of THP-1 cells in Fig. 3E was used as a control. Representative data from two independent experiments are shown.

(E-G) Effect of forced expression of caSmad3 on polyI:C-induced cell death in BT-549 cells. BT-549 cells expressing HA or caSmad3 were transfected with polyI:C (1 μg/mL). Six hours after transfection, cells were starved with serum-free culture media. After 48 hours of polyI:C transfection, the cells were analyzed with the following experiment. (E) Phase contrast micrographic images of the BT-549 cells demonstrating the effect of polyI:C transfection and expression of caSmad3. Scale bars, 200 μm. (F, G) Flow cytometric analysis of polyI:C-transfected BT-549 cells stained with annexin V-APC and PI. The percentage of annexin V-positive and PI-positive cells in FSC-gated and SSC-gated cells are shown. Representative data (F) and mean values (G) of three biological replicate samples are shown. Error bars indicate the S.D. ***P < 0.001 by Tukey-Kramer test.

(H) Effect of TGF-β pretreatment on polyI:C-induced cell death in BT-549 cells. BT-549 cells expressing HA were stimulated with TGF-β (1 ng/mL) for 24 hours before transfection of polyI:C (0.1 or 1 μg/mL). Six hours after transfection, cells were starved with serum-free culture media, and TGF-β stimulation was continued until harvesting. After 48 hours of polyI:C transfection, the cells were analyzed with flow cytometry with annexin V-APC and PI staining. The percentage of annexin V-positive and PI-positive cells in FSC-gated and SSC-gated cells with two biological replicate samples are shown.

(I) Immunoblotting of BT-549-HA or BT-549-caSmad3 cells for GSDME after 18 hours of transfection with polyI:C (1 μg/mL). Representative data from three independent experiments are shown.

**Figure S6. Mechanisms of caSmad3-mediated cell survival.**

(A) Gene expression of anti-apoptotic BCL2 family proteins in Hs578T cells quantified by RNA-seq with or without TGF-β treatment (1 ng/mL). Data are shown as the mean of two biological replicates. Error bars, S.D.

(B) Immunoblotting for phosphorylated Smad3 in Hs578T-HA cells after 1.5 hours of treatment with TGF-β (1 ng/mL) with or without 8 hours of pretreatment with TβRI inhibitor LY364947.

**Figure S7. Attenuation of p38 phosphorylation by caSmad3 partially inhibits polyI:C-induced cell death.**

(A) Immunoblotting for phosphorylated p38 in Hs578T-HA or Hs578T-caSmad3 cells after transfection of polyI:C. Twelve hours after polyI:C transfection (1 μg/mL), the cells were starved for 12 hours and then harvested. Representative data from three biological replicates are shown.

(B) Gene expression of *DUSP1* in Hs578T cells quantified by RNA-seq with or without TGF-β treatment (1 ng/mL) for 24 hours. Data obtained from two biological replicates are shown.

(C) Immunoblotting for phosphorylated HSP27 in Hs578T-HA cells after transfection with polyI:C with p38 inhibitors SB203580 and SB202190 at the indicated concentrations. The cells were pretreated with the inhibitors for 1 hour prior to polyI:C transfection (1 μg/mL). Cells were then lysed 12 hours after polyI:C transfection.

(D) Flow cytometric analysis of polyI:C-transfected Hs578T-HA cells with or without treatment with p38 inhibitors and stained with annexin V-APC and PI. Prior to polyI:C transfection, cells were treated for 1 hour with p38 inhibitors SB203580 (5 μM) and SB202190 (5 μM). Twelve hours after transfection, culture media were changed to serum-free media with or without p38 inhibitors. Cells were analyzed 36 hours after the media change. The percentage of annexin V-positive and PI-positive cells in FSC-gated and SSC-gated cells are shown. The data represent the mean of three biological replicates. Error bars, S.D.
