# Supplementary figures and images for: Anti‐pyroptotic function of TGF‐β is suppressed by a synthetic dsRNA analogue in triple negative breast cancer cells
Source: Mol Oncol. 2021 Jan 4;15(5):1289–307. doi: 10.1002/1878-0261.12890 (PMC8096786; doi:10.1002/1878-0261.12890)

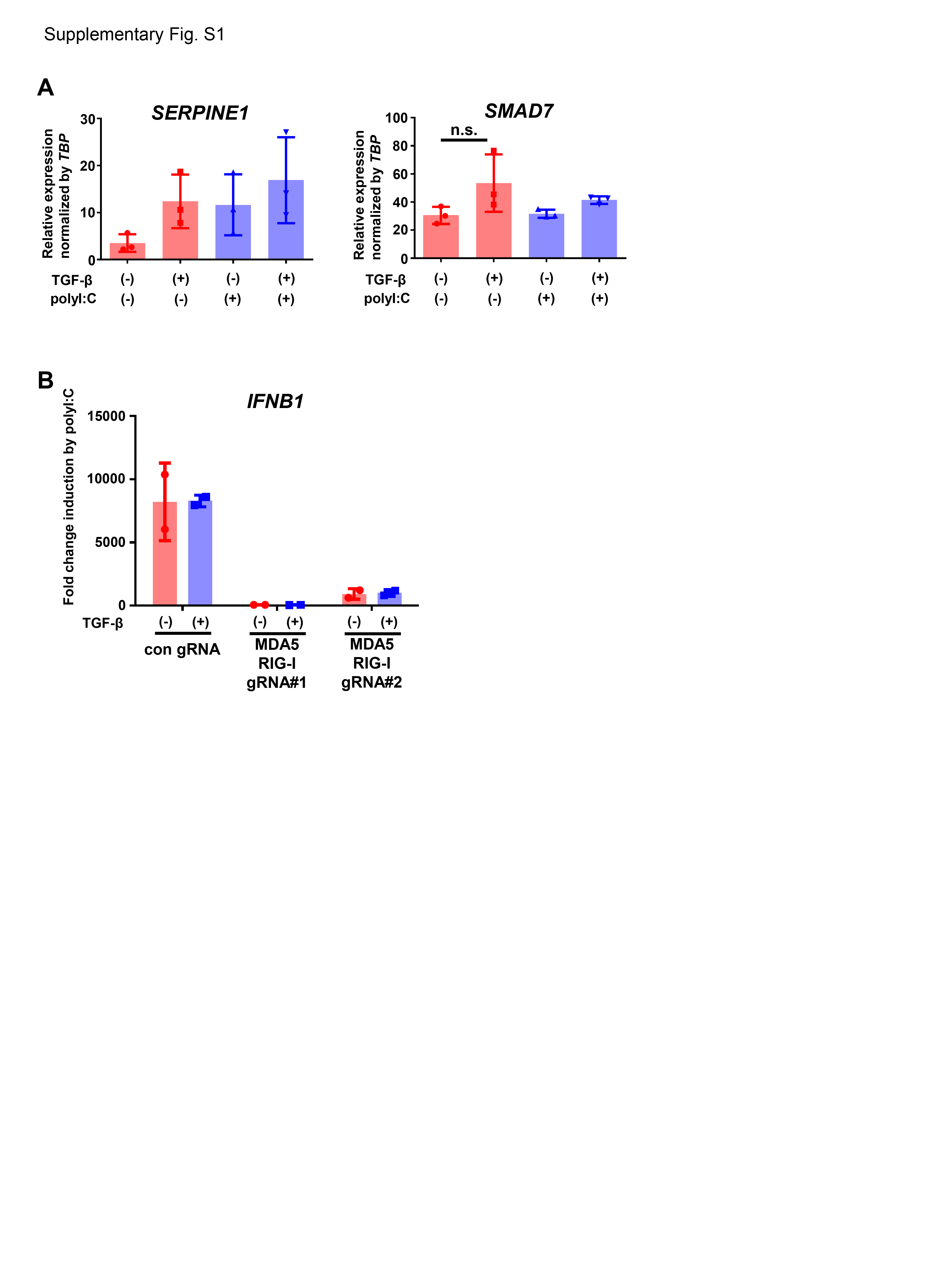

Supplement: Supplementary file 1 — Fig. S1. MDA5‐ and RIG‐I‐mediated suppression of TGF‐β signaling. Fig. S2. Potential of cell‐intrinsic activation of RLR signaling and attenuation of TGF‐β signaling. Fig. S3. caSmad3 expression in Hs578T cells and weak inhibition of polyI:C‐induced cell death by the pretreatment of TGF‐β. Fig. S4. GSDME expression in various types of cancer cells. Fig. S5. Anti‐pyroptotic effect of TGF‐β is suppressed by polyI:C in BT‐549 cells. Fig. S6. Mechanisms of caSmad3‐mediated cell survival. Fig. S7. Attenuation of p38 phosphorylation by caSmad3 partially inhibits polyI:C‐induced cell death. [file MOL2-15-1289-s004.zip › mol212890-sup-0001-FigS1.tif]

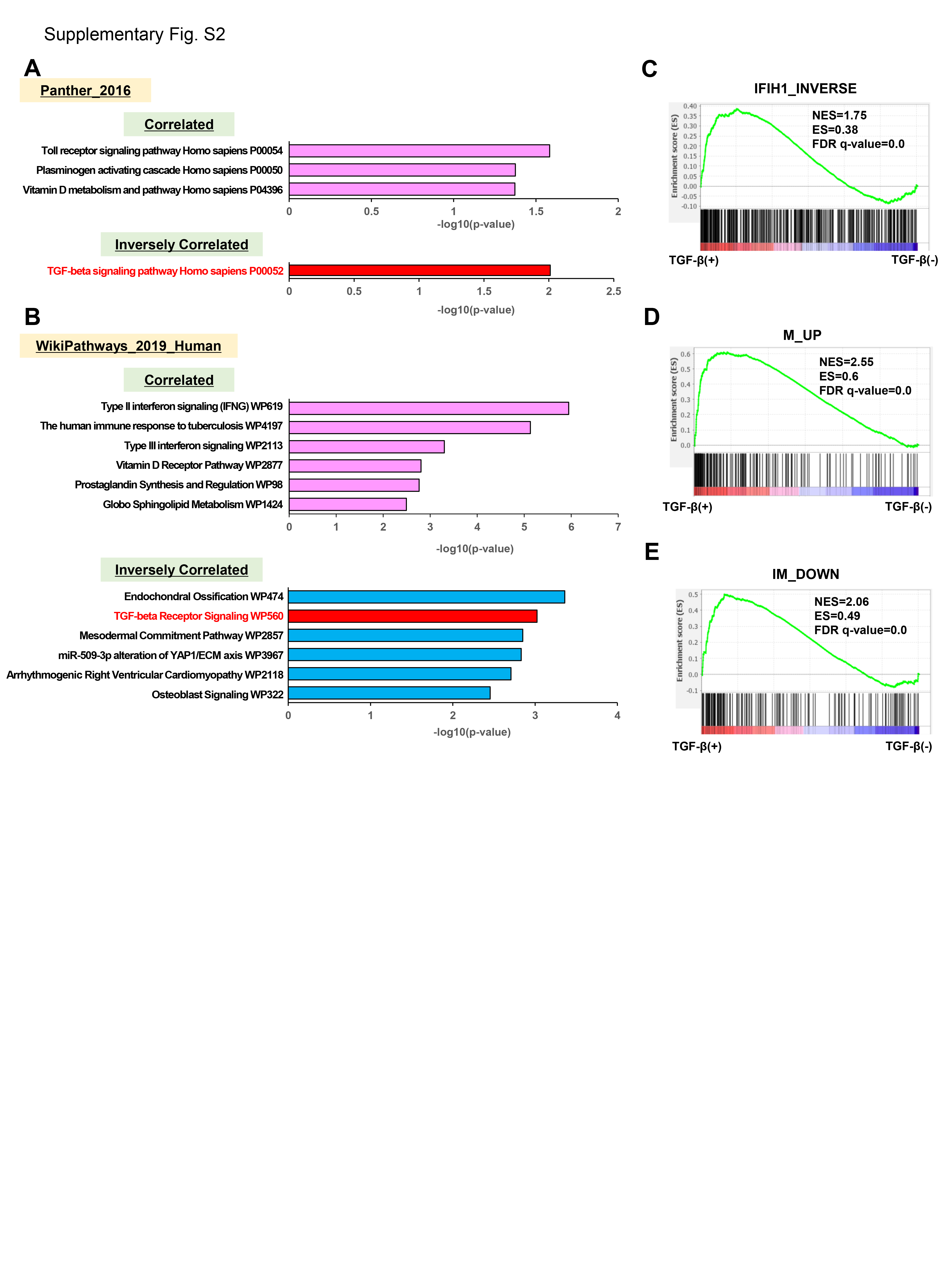

Supplement: Supplementary file 1 — Fig. S1. MDA5‐ and RIG‐I‐mediated suppression of TGF‐β signaling. Fig. S2. Potential of cell‐intrinsic activation of RLR signaling and attenuation of TGF‐β signaling. Fig. S3. caSmad3 expression in Hs578T cells and weak inhibition of polyI:C‐induced cell death by the pretreatment of TGF‐β. Fig. S4. GSDME expression in various types of cancer cells. Fig. S5. Anti‐pyroptotic effect of TGF‐β is suppressed by polyI:C in BT‐549 cells. Fig. S6. Mechanisms of caSmad3‐mediated cell survival. Fig. S7. Attenuation of p38 phosphorylation by caSmad3 partially inhibits polyI:C‐induced cell death. [file MOL2-15-1289-s004.zip › mol212890-sup-0002-FigS2.tif]

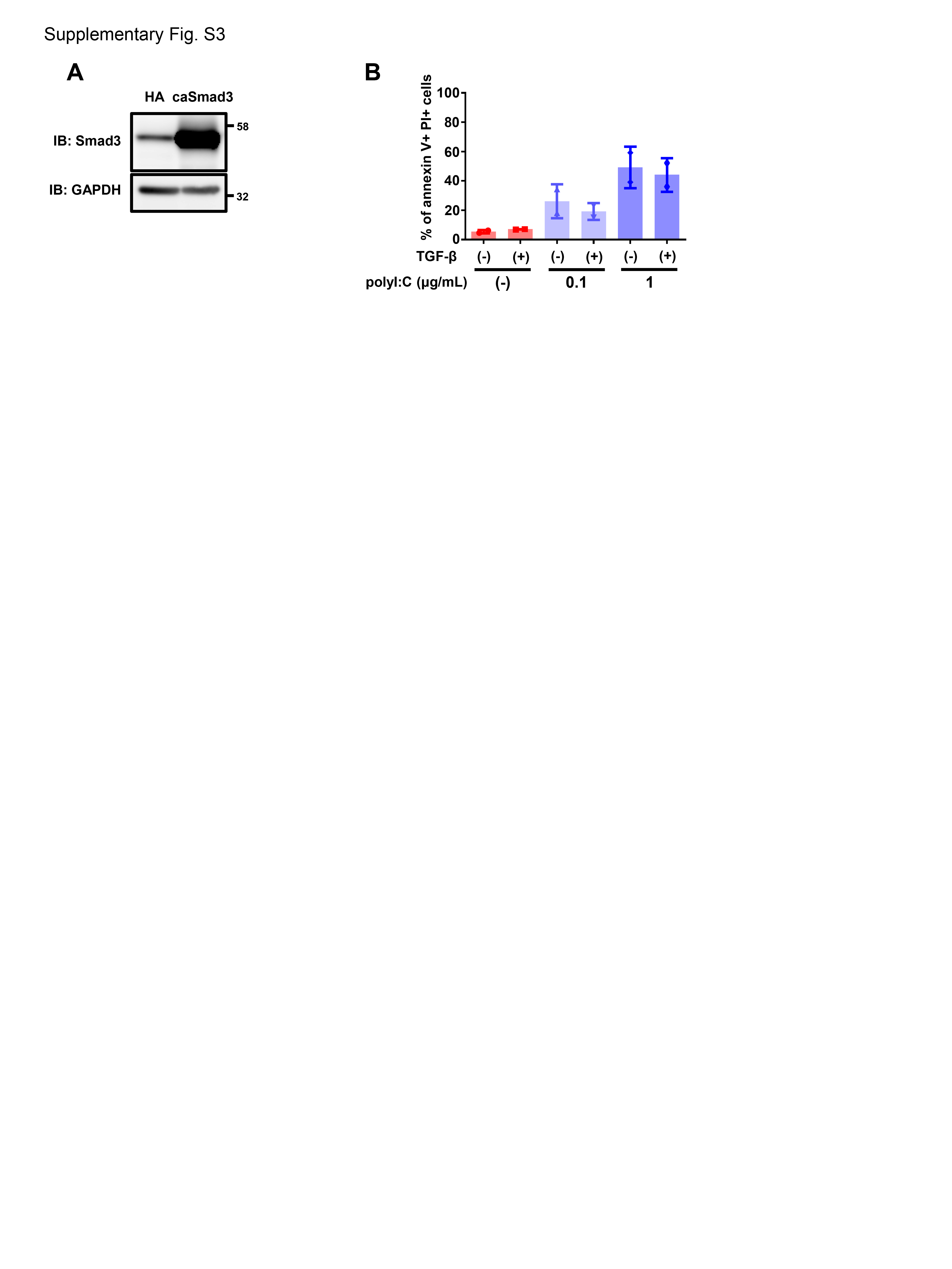

Supplement: Supplementary file 1 — Fig. S1. MDA5‐ and RIG‐I‐mediated suppression of TGF‐β signaling. Fig. S2. Potential of cell‐intrinsic activation of RLR signaling and attenuation of TGF‐β signaling. Fig. S3. caSmad3 expression in Hs578T cells and weak inhibition of polyI:C‐induced cell death by the pretreatment of TGF‐β. Fig. S4. GSDME expression in various types of cancer cells. Fig. S5. Anti‐pyroptotic effect of TGF‐β is suppressed by polyI:C in BT‐549 cells. Fig. S6. Mechanisms of caSmad3‐mediated cell survival. Fig. S7. Attenuation of p38 phosphorylation by caSmad3 partially inhibits polyI:C‐induced cell death. [file MOL2-15-1289-s004.zip › mol212890-sup-0003-FigS3.tif]

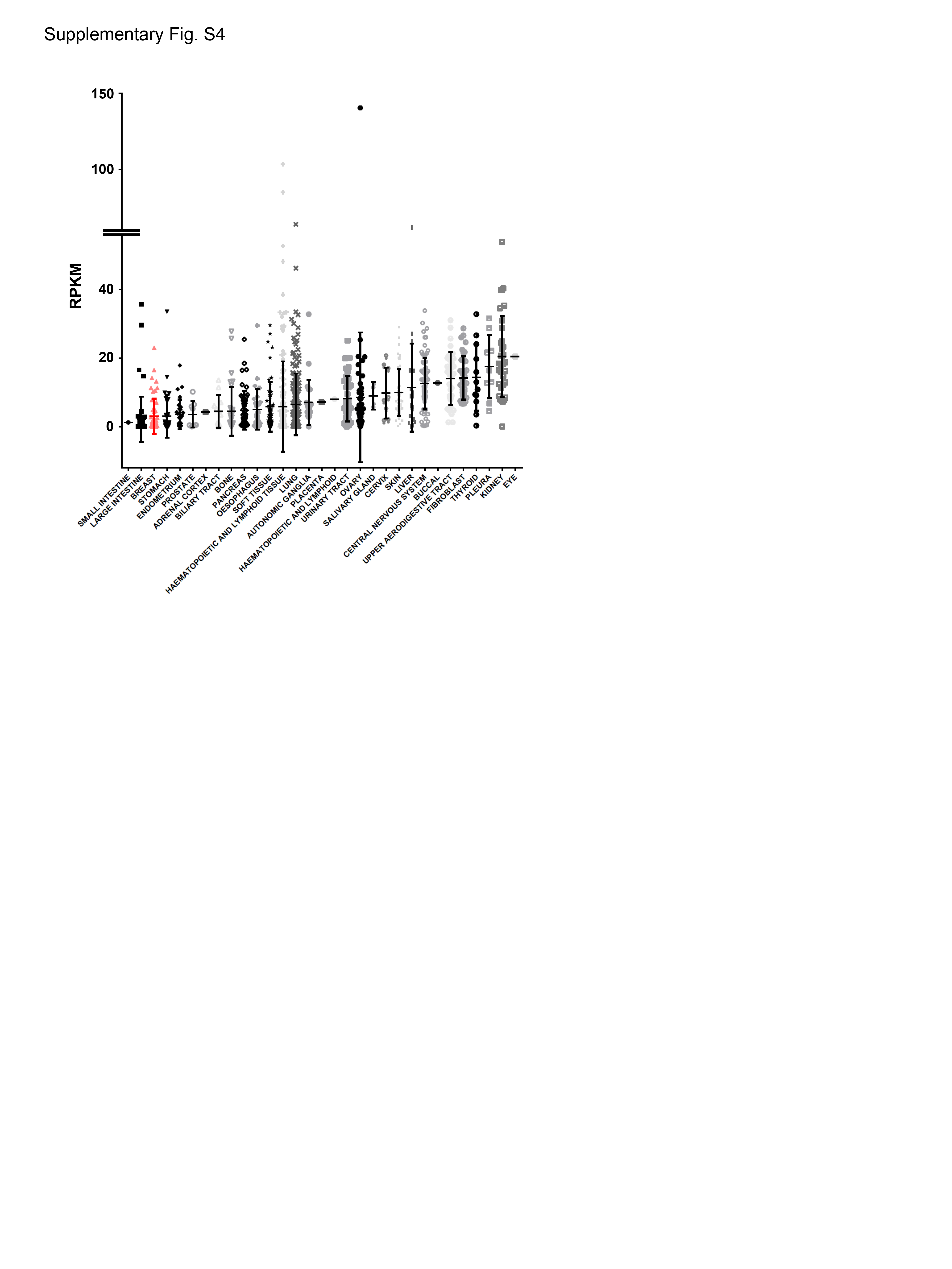

Supplement: Supplementary file 1 — Fig. S1. MDA5‐ and RIG‐I‐mediated suppression of TGF‐β signaling. Fig. S2. Potential of cell‐intrinsic activation of RLR signaling and attenuation of TGF‐β signaling. Fig. S3. caSmad3 expression in Hs578T cells and weak inhibition of polyI:C‐induced cell death by the pretreatment of TGF‐β. Fig. S4. GSDME expression in various types of cancer cells. Fig. S5. Anti‐pyroptotic effect of TGF‐β is suppressed by polyI:C in BT‐549 cells. Fig. S6. Mechanisms of caSmad3‐mediated cell survival. Fig. S7. Attenuation of p38 phosphorylation by caSmad3 partially inhibits polyI:C‐induced cell death. [file MOL2-15-1289-s004.zip › mol212890-sup-0004-FigS4.tif]

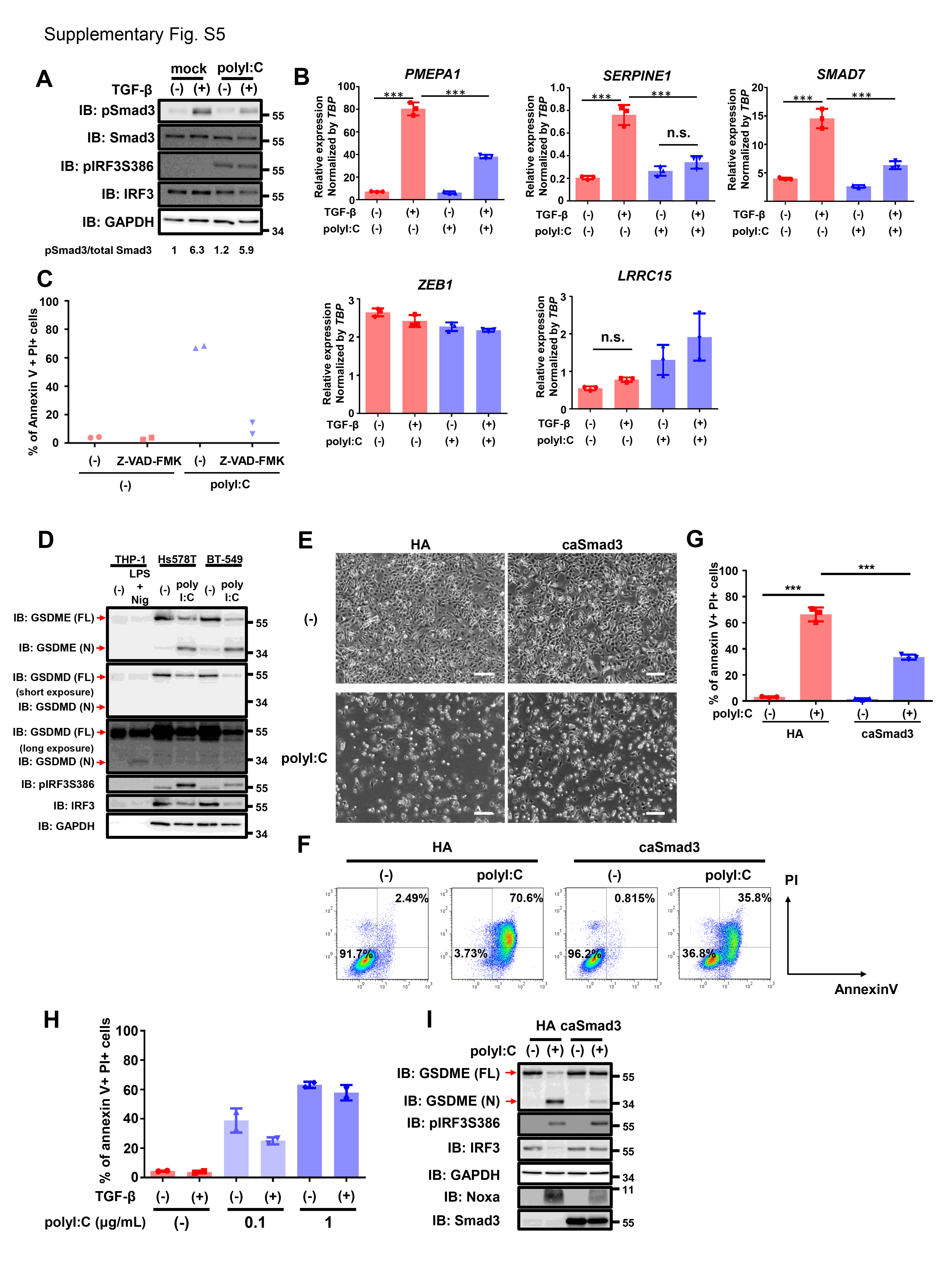

Supplement: Supplementary file 1 — Fig. S1. MDA5‐ and RIG‐I‐mediated suppression of TGF‐β signaling. Fig. S2. Potential of cell‐intrinsic activation of RLR signaling and attenuation of TGF‐β signaling. Fig. S3. caSmad3 expression in Hs578T cells and weak inhibition of polyI:C‐induced cell death by the pretreatment of TGF‐β. Fig. S4. GSDME expression in various types of cancer cells. Fig. S5. Anti‐pyroptotic effect of TGF‐β is suppressed by polyI:C in BT‐549 cells. Fig. S6. Mechanisms of caSmad3‐mediated cell survival. Fig. S7. Attenuation of p38 phosphorylation by caSmad3 partially inhibits polyI:C‐induced cell death. [file MOL2-15-1289-s004.zip › mol212890-sup-0005-FigS5.tif]

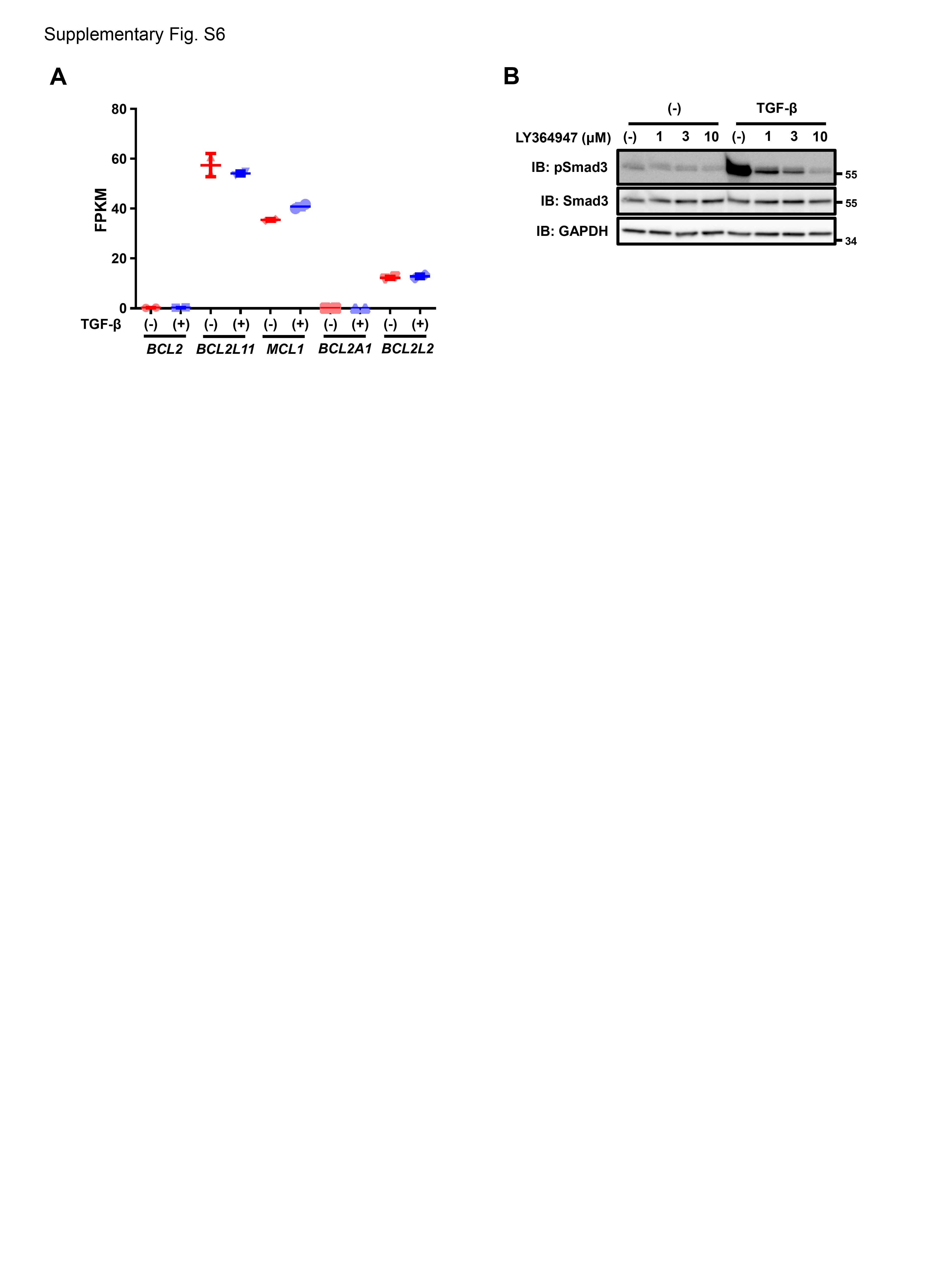

Supplement: Supplementary file 1 — Fig. S1. MDA5‐ and RIG‐I‐mediated suppression of TGF‐β signaling. Fig. S2. Potential of cell‐intrinsic activation of RLR signaling and attenuation of TGF‐β signaling. Fig. S3. caSmad3 expression in Hs578T cells and weak inhibition of polyI:C‐induced cell death by the pretreatment of TGF‐β. Fig. S4. GSDME expression in various types of cancer cells. Fig. S5. Anti‐pyroptotic effect of TGF‐β is suppressed by polyI:C in BT‐549 cells. Fig. S6. Mechanisms of caSmad3‐mediated cell survival. Fig. S7. Attenuation of p38 phosphorylation by caSmad3 partially inhibits polyI:C‐induced cell death. [file MOL2-15-1289-s004.zip › mol212890-sup-0006-FigS6.tif]

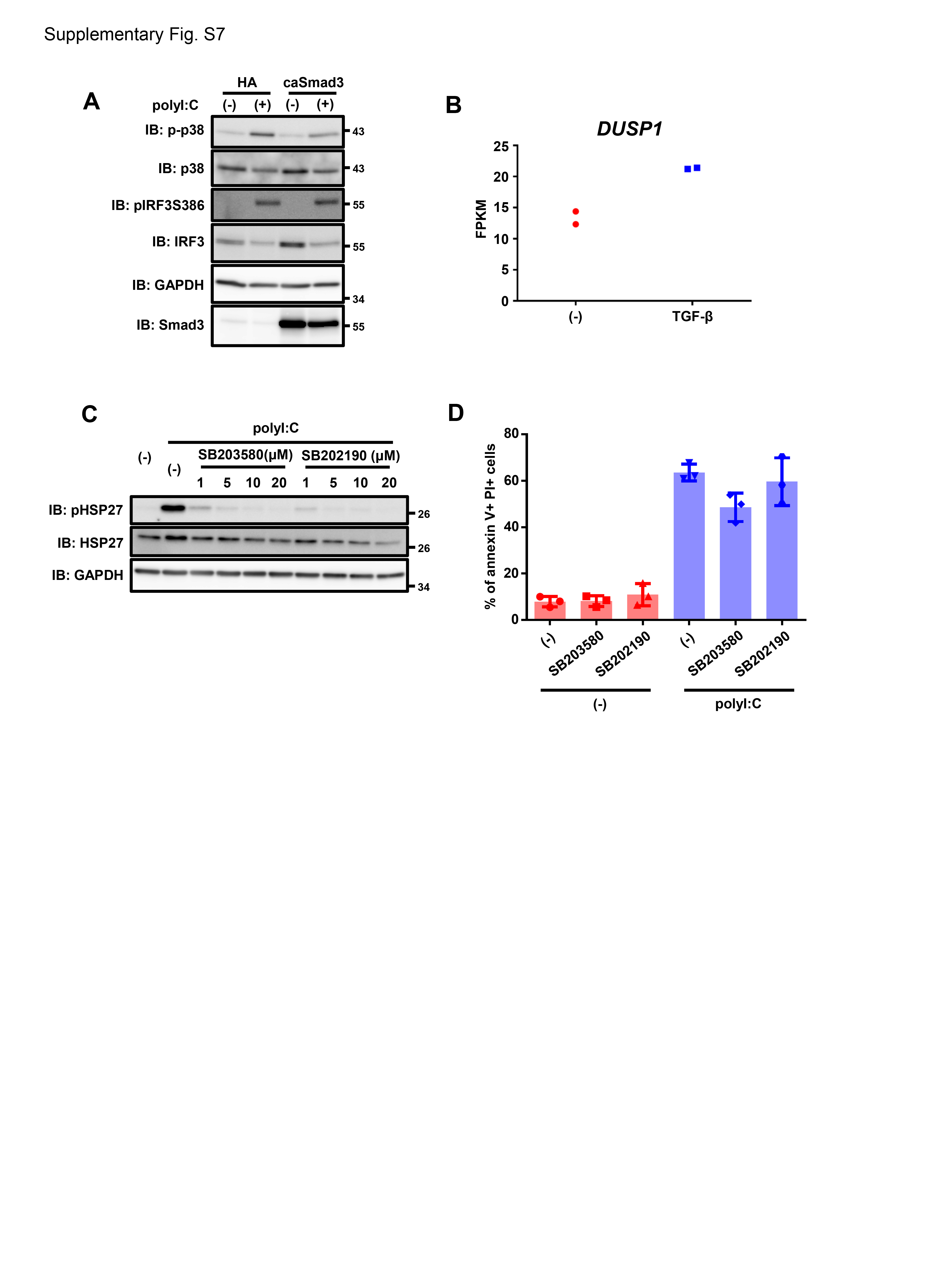

Supplement: Supplementary file 1 — Fig. S1. MDA5‐ and RIG‐I‐mediated suppression of TGF‐β signaling. Fig. S2. Potential of cell‐intrinsic activation of RLR signaling and attenuation of TGF‐β signaling. Fig. S3. caSmad3 expression in Hs578T cells and weak inhibition of polyI:C‐induced cell death by the pretreatment of TGF‐β. Fig. S4. GSDME expression in various types of cancer cells. Fig. S5. Anti‐pyroptotic effect of TGF‐β is suppressed by polyI:C in BT‐549 cells. Fig. S6. Mechanisms of caSmad3‐mediated cell survival. Fig. S7. Attenuation of p38 phosphorylation by caSmad3 partially inhibits polyI:C‐induced cell death. [file MOL2-15-1289-s004.zip › mol212890-sup-0007-FigS7.tif]
